# Supplementary material for: Wildfires on a changing planet
Source: Nat Commun. 2026 Feb 12;17:1599. doi: 10.1038/s41467-025-68176-4 (PMC12905353; doi:10.1038/s41467-025-68176-4)
Supplement: Supplementary file 1 — Supplementary Information [file 41467_2025_68176_MOESM1_ESM.pdf]

Supplementary information: Wildfires on a Changing Planet

Supplementary Section 1: BA inter-annual variability performance and trends

The modelled mean annual burnt area from 2002-2019 was 755 Mha, intermediate between the values of 418 Mha for the MODIS BA (107) and 769 Mha for the GFED5 (108) burnt area data products. The model captured the global and regional observed year-to-year variability in global burnt area reasonably well however it failed to capture the declining global trend over the period of 2002 to 2019 (See Fig. S1 and Fig S2). We investigated what caused this divergence in the predicted and observed trend but conducting four sensitivity analyses: 1) holding GPP, GPP seasonality, tree, shrub and grass constant over the period (GPP and natural vegetation sensitivity) and varying all other predictors, 2) holding DD, DD seasonality, VPD, DTR and wind constant over the period (Climate sensitivity) and varying all other predictors, 3) holding everything constant expect for cropland cover, road density and population density (GPP, natural vegetation and climate sensitivity) and 4) varying only cropland cover, road density and population density (human activity sensitivity) and holding all other predictors constant(see Fig. S1, Fig. S2 and Table S1). A linear regression was performed to assess these trends.

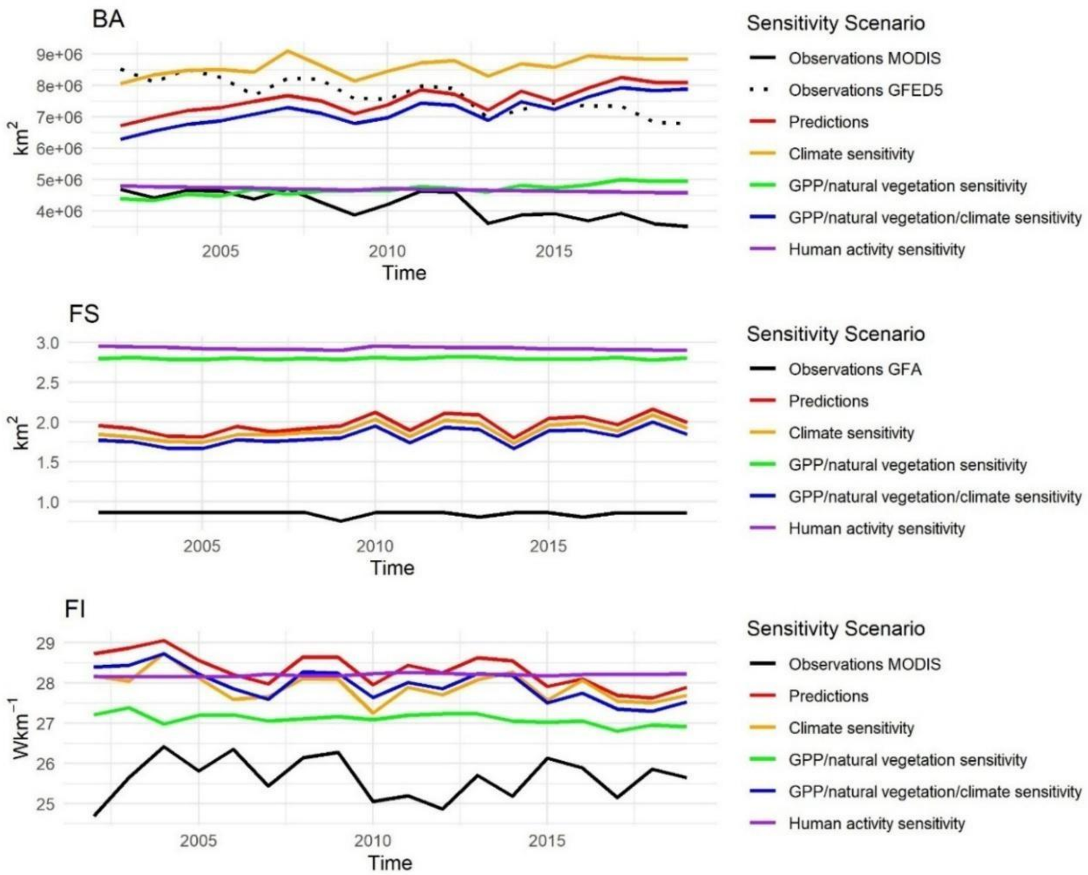

Supplementary Figure S1. Contemporary trends in fire regimes. Global 18-year trend in burnt area (BA; top panel) and fire size (FS; top panel) in km^2 and fire intensity (FI; bottom panel) in in km^{-1} from 2002-2019. Predicted trends where all predictors where varying are shown in red, and observations are shown in black. The trend in the GPP and natural vegetation sensitivity run is shown in green, the trend in the Climate sensitivity analysis is shown in orange, the trend in the GPP and natural vegetation and climate sensitivity run is shown in dark green and the trend in the human activity sensitivity analysis is shown in purple.

Supplementary Table S1. NME scores (109) of trends in interannual variability (step 2) for modelled burnt area against FIRMS MODIS and GFED5 for the 2002 to 2019 period. *** represents a $p < 0.001$ significance level, ** represents a $p < 0.01$ significance level and * represents a $p < 0.05$ significance level.

	Burnt Area			Fire Size		Fire Intensity	
Observational Product	Predicted	MODIS	GFED5	Predicted	GFA	Predicted	MODIS
	Trend (km^2)	NME	NME	Trend (m^2)	NME	Trend (W.km-1)	NME
All predictors	176.86***	1.51	1.45	0.003*	0.87	-0.0002***	1.10
Fragmentation and human activity only	-248.72***	0.87	0.91	0	1.11	0	1.00
Climate predictors only	83.74***	1.17	1.15	0.003*	1.81	0	0.90
GPP and natural vegetation only	87.86***	1.17	1.25	0	1.00	-0.00004**	1.00

The trends in fire size and fire intensity are close to zero due to the distribution of these variables, with many grid-cells with very small values of median fire size and fire intensity. As such, the trends in the mean global values are very small but remain significant and are rounded to zero in the table. The trend in fire size was best when all changes in all predictors were applied and the trend in fire intensity was best when only changes in climate predictors were applied. For burnt area, when only changes in human activity are considered, a negative trend of $-248.72\ km^2\ yr^{-1}$ is observed whilst when only climate changes are considered, a positive trend of $+83.74\ km^2\ yr^{-1}$ is observed. Over the 18-year period this corresponds to a total modelled decline of ~4.5 Mha from human activity and an increase of ~1.5 Mha from climate. It is worth noting that the absolute magnitudes of these trends are about an order of magnitude smaller than observed, likely reflecting simplifications in how the model represents fire–human–climate interactions. Nevertheless, the relative balance between human- and climate-driven trends is consistent with independent analyses. The sensitivity analysis gives a relative balance of roughly 3:1, consistent with the literature, which suggests a relative balance of ~2.5:1. This balance can be derived from the ~−25% decrease in burned area from human drivers over 18 years cited in Andela et al., 2017 and the ~+16% increase from climate over 2003-2019 cited in Burton et al., 2024. Despite underestimating their strength, the relative trends in the human and non-human predictors of this model are plausible and align with previous work (7,4,21Andela et al., 2017; Jones et al., 2022; Burton et al., 2024).

Supplementary Table S2. Interannual-trends in burnt area and NME scores (step 2) of the against observations for the original model.

	Original BA		
GFED Region	Predicted trend (km^2-yr^{-1})	NME step 2 (GFED5)	NME step 2 (MODIS)
BONA	1047384.58	1.37	1.52
TENA	35.19	4.10	4.70
CEAM	1269593.78	1.10	1.87
NHSA	37.89	1.61	1.76
SHSA	108078.38	1.48	1.53
EURO	11.33	1.91	3.36
MIDE	116160.18	0.80	1.08
NHAF	9.55	1.15	1.27
SHAF	214827.38	2.14	1.64
BOAS	1.53	1.18	1.33
CEAS	95469.28	1.11	1.12
SEAS	-0.49	0.90	1.03
EQAS	477143.27	0.48	0.58
AUST	17.95	0.94	1.00

Supplementary Section 2: Realistic experiments when considering grid-cell level tree, shrub and grass cover

In this analysis we consider differences between vegetation types only through the distinction of fractional tree, grass and shrub cover. These fractions are obtained by averaging values for each biome globally. We therefore do not consider within-biome responses, and this could potentially explain some regional differences. However, there are reasons to expect that little information is thereby lost. Research has consistently shown that current biomes are not representative of differences in fire properties and that other plant traits which are not captured in this classification, but that relate to flammability and recovery, are more important (Belcher et al., 2018; Harrison et al., 2021). Moreover, predictors relating to vegetation amount have been shown to be more important in capturing burnt area than predictors relating to vegetation type (Haas et al., 2024). Our vegetation productivity estimates are independent of plant functional type parameters and do allow us to observe regional differences between regions with similar conditions, reducing the uncertainty introduced by fixed vegetation categories.

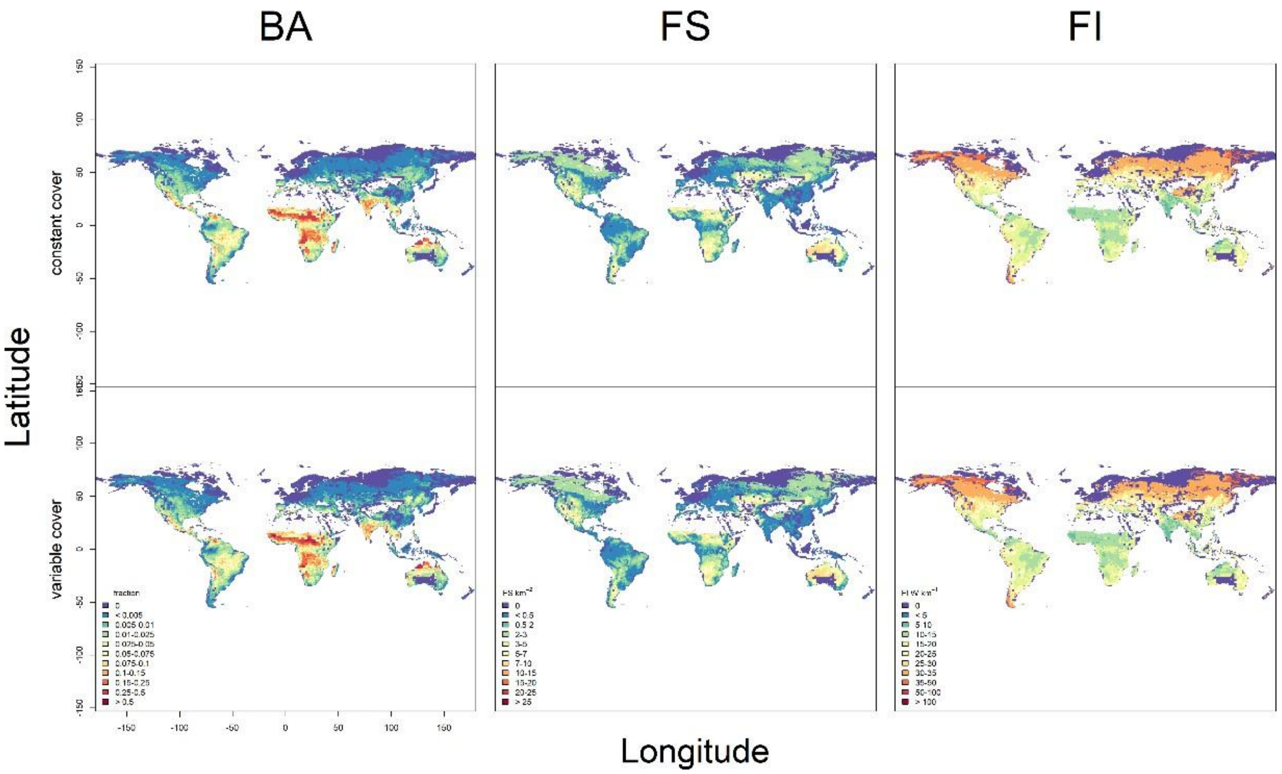

Supplementary Fig S2. Predicted fire properties with constant land cover. Burnt area (BA; first column), fire size (FS; second column) and fire intensity (FI; third column) simulated using constant grass, tree and shrub cover within each BIOME4 biome (top panel) and using variable grass, tree and shrub cover within each BIOME4 biome (bottom panel).

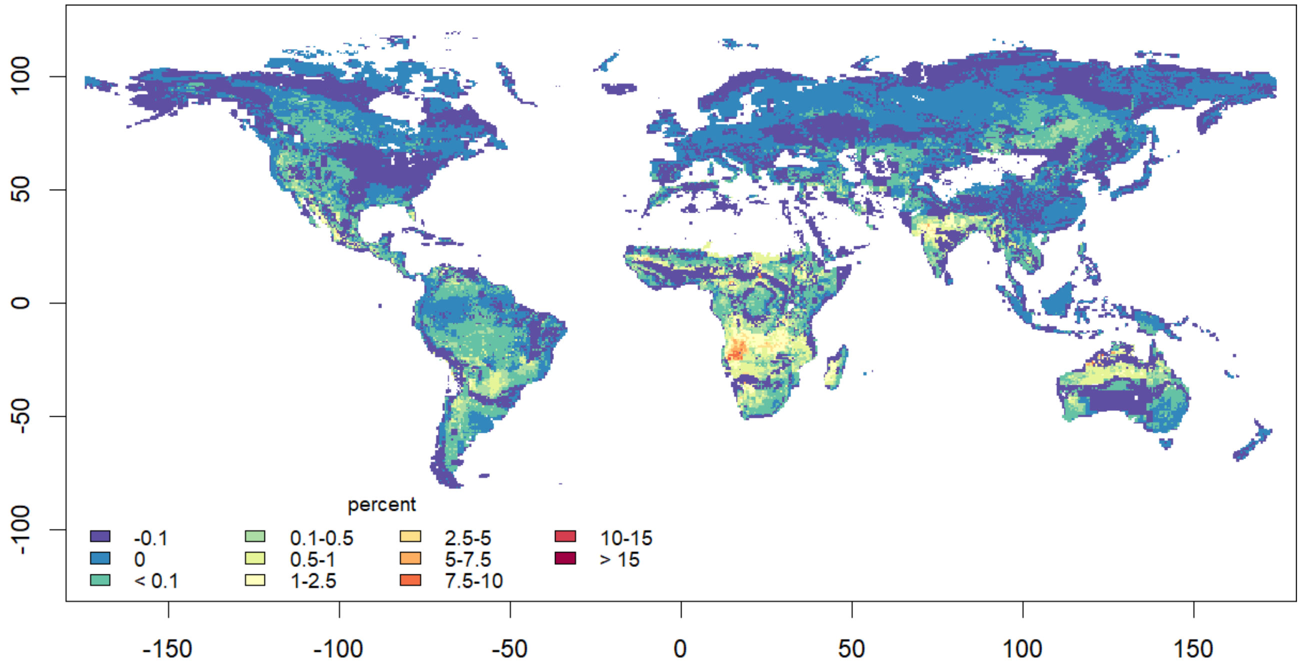

Supplementary Fig S3. Percentage difference in burnt area due to land cover. The percentage difference in modelled burnt area (BA) between constant tree, shrub and grass cover within each biome and varying tree, shrub and grass cover within each biome.

Supplementary Table S3. Global burnt area (M km^2), fire size (km^2) and fire intensity (km^{-1}) using ESA landcover, BIOME4 simulated constant land cover and BIOME4 simulated varying land cover, along with the percentage change between the modern and end-of-century simulations for RCP2.6 and RCP6.0.

	Modern	Percentage change RCP2.6	Percentage change RCP6.0
Burnt Area			
ESA	4.44		
Constant	3.96	-11.39	-9.20
Variable	3.81	27.68	29.08
Fire Size			
ESA	2.59		
Constant	3.86	27.46	20.59
Variable	2.80	29.53	22.23
Fire Intensity			
ESA	31.25		
Constant	26.54	5.87	-2.30
Variable	26.30	5.19	-2.15

Supplementary Section 3: Realistic experiments

All figures presented below are from the realistic experiments where changes occurred in climate inputs, CO_2 levels (affecting vegetation) and human activity under high (SPP2-RCP2.6) and low (SSP2-RCP6.0) climate change mitigation efforts. In addition to the results discussed in the main text, this section of the supplementary provides more detailed information on the global results from each individual climate model (Table S4) and the global anomalies for the mean of the four models (FigS9). It also provides a more detailed breakdown of the trends between additional biome types (Fig S10) as well as global maps of the most important variable in driving change in the fire properties under modern and scenario conditions (Fig S15-S18).

Supplementary Table S4. Global annual burnt area (BA), mean fire size (FS) and mean fire intensity (FI) values for the realistic experiments.

BA (Mkm^2)	Modern-day (realistic)	High climate change mitigation	Percentage change	Low climate change mitigation	Percentage change
GFDL-ESM2M	4.06 [3.86-4.27]	3.05 [2.88-3.23]	-24.89 [-25.52--24.25]	6.1 [5.74-6.49]	50.31 [48.63-52.01]
IPSL-CM5A-LR	4.06 [3.86-4.27]	3.9 [3.69-4.13]	-3.84 [-4.53--3.15]	6.39 [6.01-6.8]	57.46 [55.66-59.23]
HadGEM2-ES	4.06 [3.86-4.27]	3.24 [3.05-3.44]	-20.19 [-20.94--19.42]	6.49 [6.09-6.91]	59.81 [57.72-61.88]
MIROC5	4.06 [3.86-4.27]	3.23 [3.05-3.43]	-20.34 [-21.01--19.66]	4.62 [4.34-4.92]	13.9 [12.46-15.35]
FS (km^2)	Modern-day (realistic)	High climate change mitigation	Percentage change	Low climate change mitigation	Percentage change
GFDL-ESM2M	2.84 [2.72-2.96]	3.4 [3.24-3.55]	19.73 [19.23-19.68]	3.67 [3.49-3.85]	29.29 [28.29-29.89]
IPSL-CM5A-LR	2.84 [2.72-2.96]	3.67 [3.51-3.83]	29.24 [29.08-29.12]	3.6 [3.44-3.78]	27.06 [26.4-27.56]
HadGEM2-ES	2.84 [2.72-2.96]	3.74 [3.59-3.89]	31.9 [32.16-31.43]	3.29 [3.14-3.44]	15.93 [15.46-16.25]
MIROC5	2.84 [2.72-2.96]	3.76 [3.61-3.92]	32.67 [32.83-32.18]	3.62 [3.47-3.78]	27.67 [27.57-27.52]
FI ($W.km^{-1}$)	Modern-day (realistic)	High climate change mitigation	Percentage change	Low climate change mitigation	Percentage change
GFDL-ESM2M	26.42 [25.6-27.33]	27.45 [26.68-28.24]	3.86 [4.21-3.34]	25.24 [24.61-25.88]	-4.49 [-3.87--5.32]
IPSL-CM5A-LR	26.42 [25.6-27.33]	27.16 [26.51-27.84]	2.77 [3.54-1.87]	24.78 [24.15-25.46]	-6.21 [-5.64--6.86]
HadGEM2-ES	26.42 [25.6-27.33]	27.91 [27.24-28.62]	5.63 [6.4-4.7]	23.13 [22.49-23.83]	-12.48 [-12.14--12.82]
MIROC5	26.42 [25.6-27.33]	27.91 [27.23-28.62]	5.64 [6.37-4.73]	26.25 [25.59-26.97]	-0.65 [-0.02--1.32]

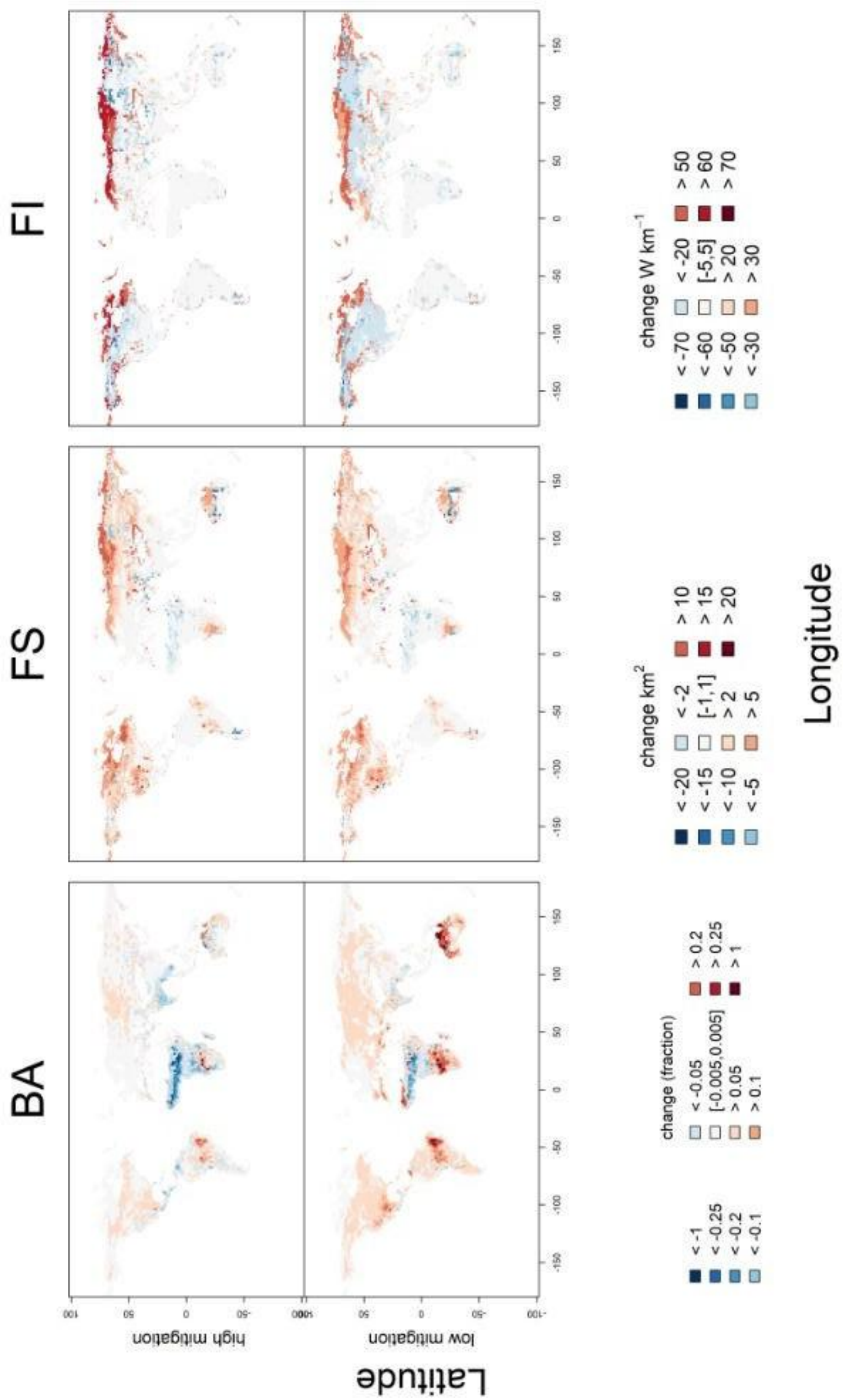

Supplementary Fig S4. Fire properties anomalies. Anomalies for burnt area (BA; first column), fire size (FS; second column) and fire intensity (FI; third column) between the modern-day experiment and the high climate change mitigation experiment (top panel) and low climate change mitigation experiment (bottom panel) for the mean of the experiments driven by the outputs of all four climate models (mean) in which CO_2 levels are 424ppm and 651ppm respectively and human activity follows a SSP2 trajectory. Red represents an increase in the fire property by the end of the century, and blue represents a decrease.

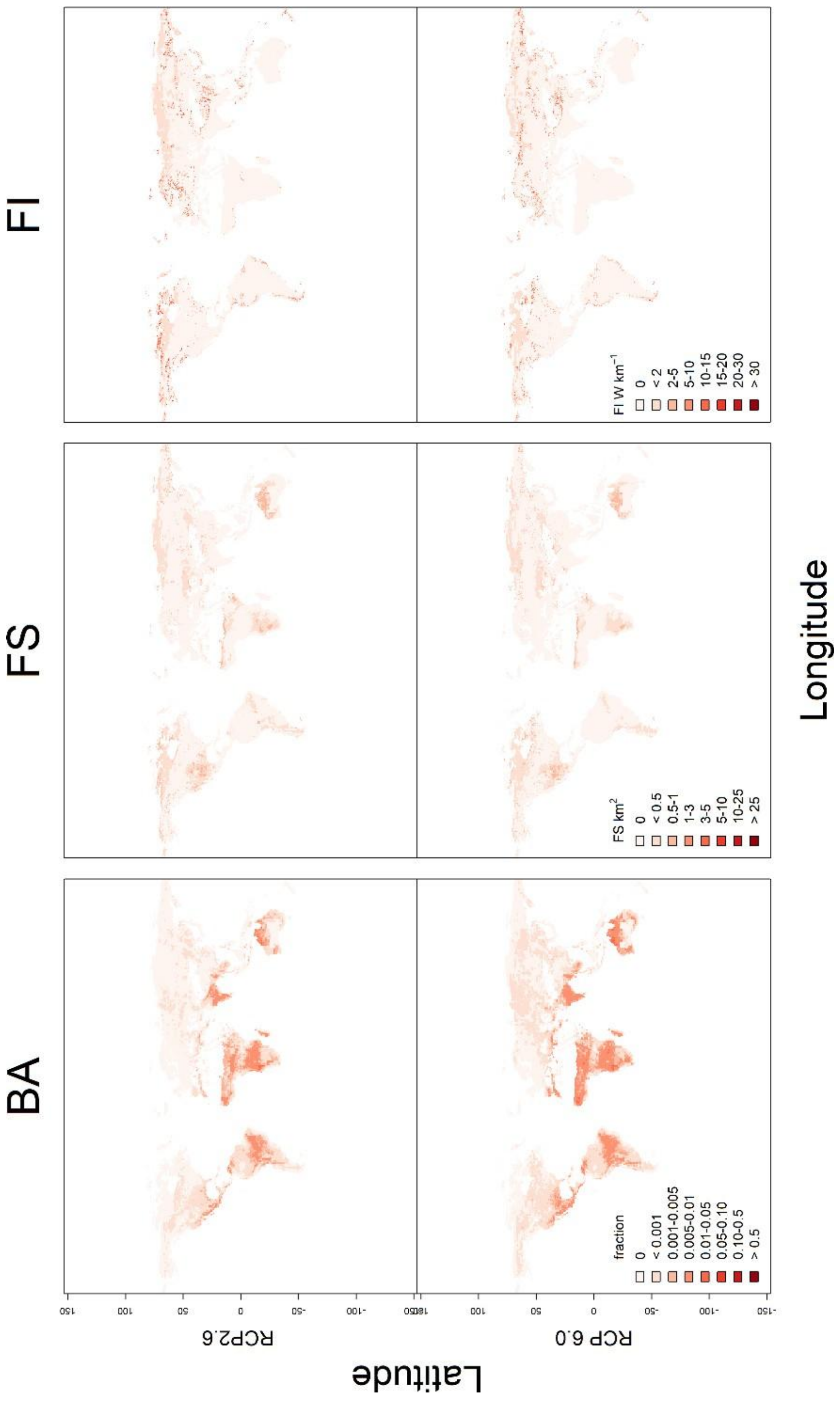

Supplementary Fig S5. Spread in fire properties predictions. Spread in prediction interval burnt area (BA; first column), fire size (FS; second column) and fire intensity (FI; third column) between the modern-day experiment and the high climate change mitigation experiment (top panel) and low climate change mitigation experiment (bottom panel) for the mean of the experiments driven by the outputs of all four climate models (mean) in which CO_2 levels are 424ppm and 651ppm respectively and human activity follows a SSP2 trajectory. Red represents an increase in the fire property by the end of the century, and blue represents a decrease.

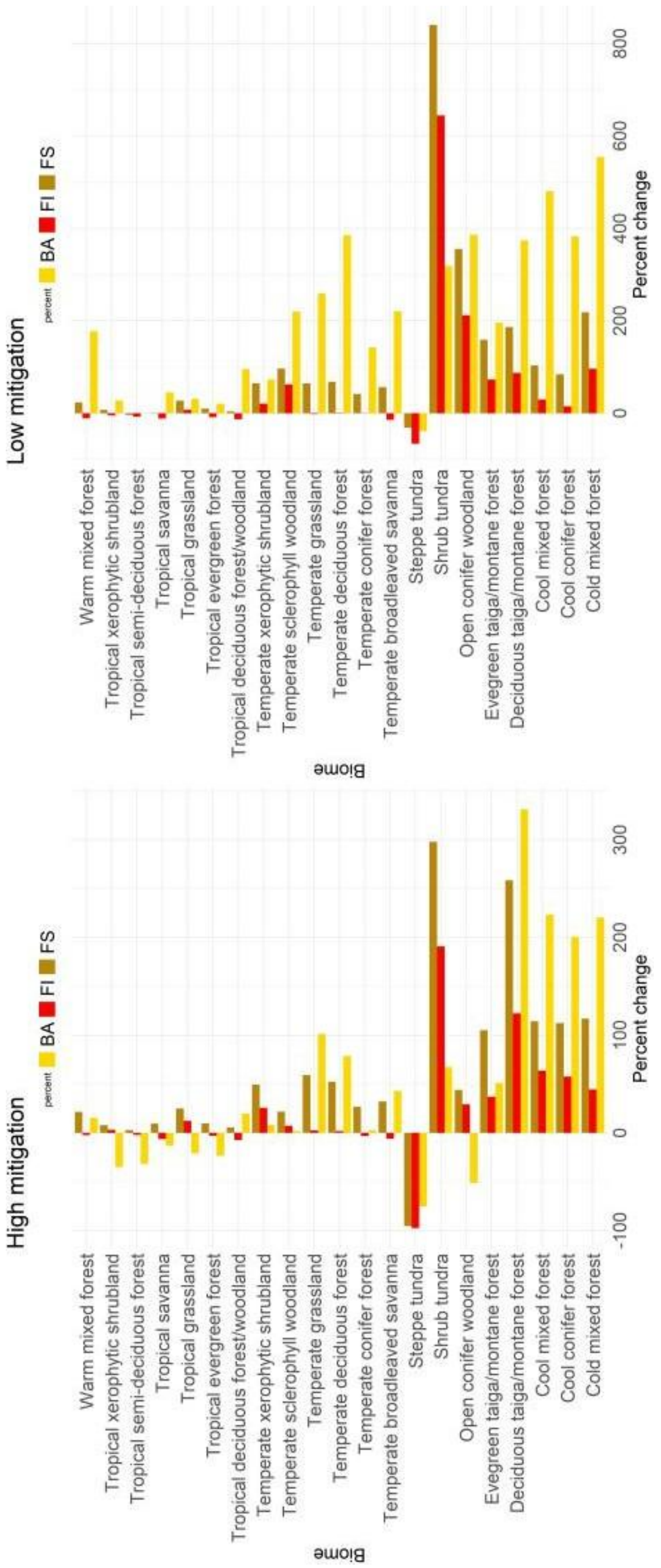

Supplementary Fig S6. Biome breakdown of predicted change. Percentage change for all three fire properties between realistic modern-day conditions and the realistic future experiments broken down by biome type. The mean is across all four of the climate model experiments for each scenario. BA in red, FS in brown and FI in red.

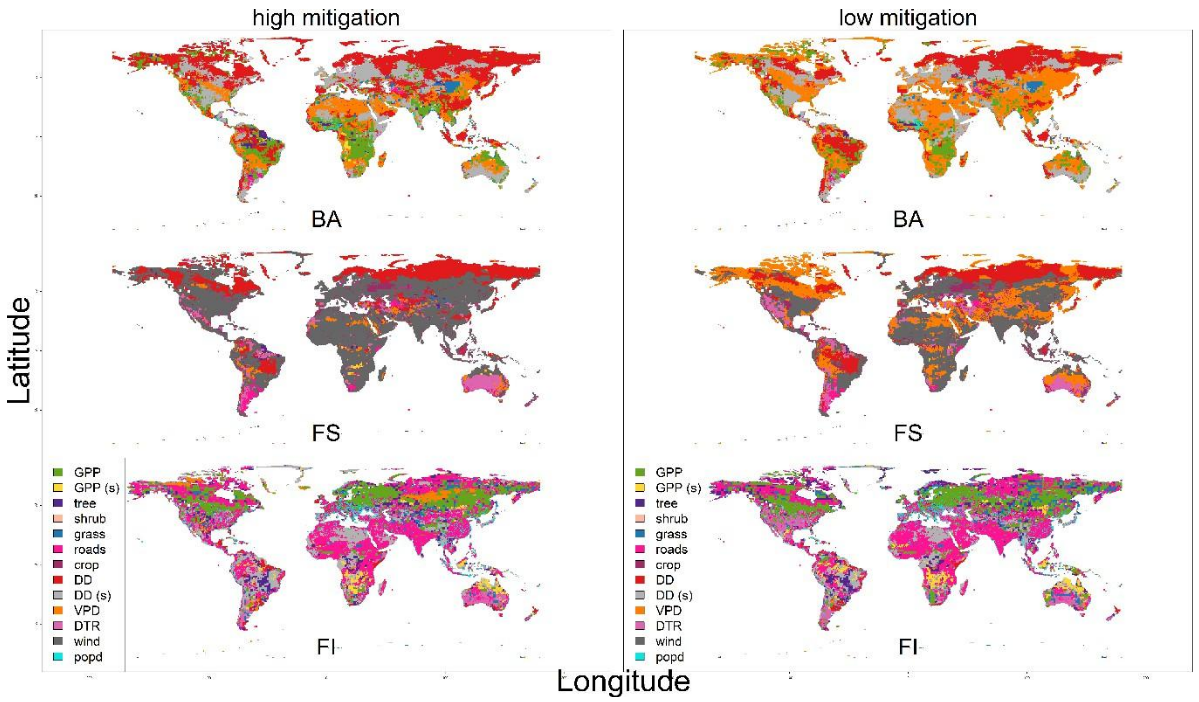

Supplementary Fig S7. Positive variable contribution for GFDL-ESM2M. Map showing for each grid-cell which variable in the GLM model caused the positive contribution in the future projections for the high climate change mitigation (left) and low climate change mitigation (right) experiment for the GFDL-ESM2M climate model outputs for BA (first row), FS (second row) and FI (third row).

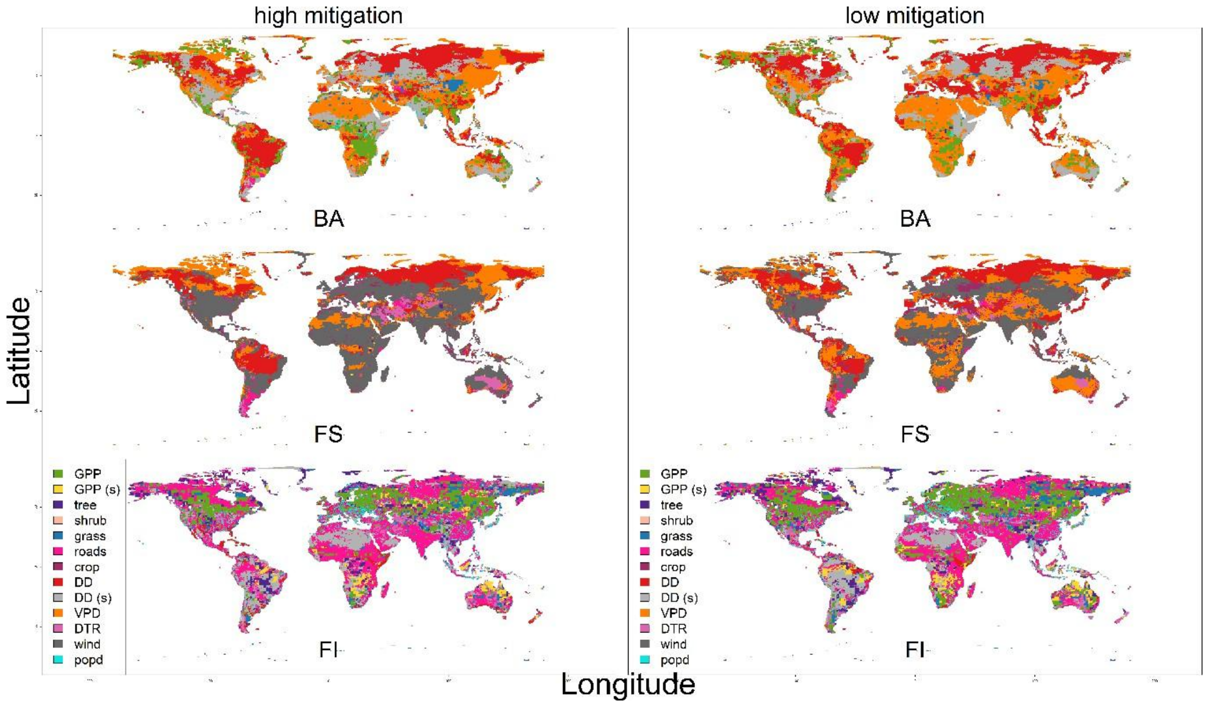

Supplementary Fig S8. Positive variable contribution of IPSL-CM5A-LR. Map showing for each grid-cell which variable in the GLM model caused the positive contribution in the future projections for the high climate change mitigation (left) and low climate change mitigation (right) experiment for the IPSL-CM5A-LR climate model outputs for BA (first row), FS (second row) and FI (third row).

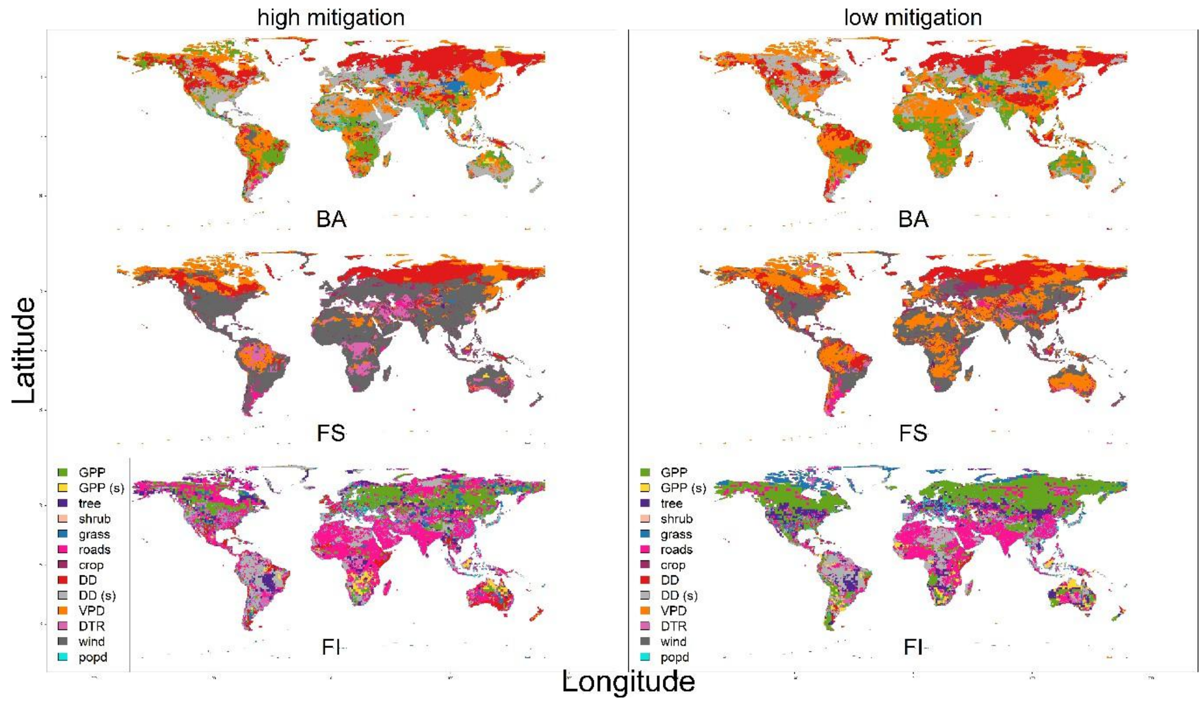

Supplementary Fig S9. Positive variable contribution for Had-GEM2-ES. Map showing for each grid-cell which variable in the GLM model caused the positive contribution in the future projections for the high climate change mitigation (left) and low climate change mitigation (right) experiment for the Had-GEM2-ES climate model outputs for BA (first row), FS (second row) and FI (third row).

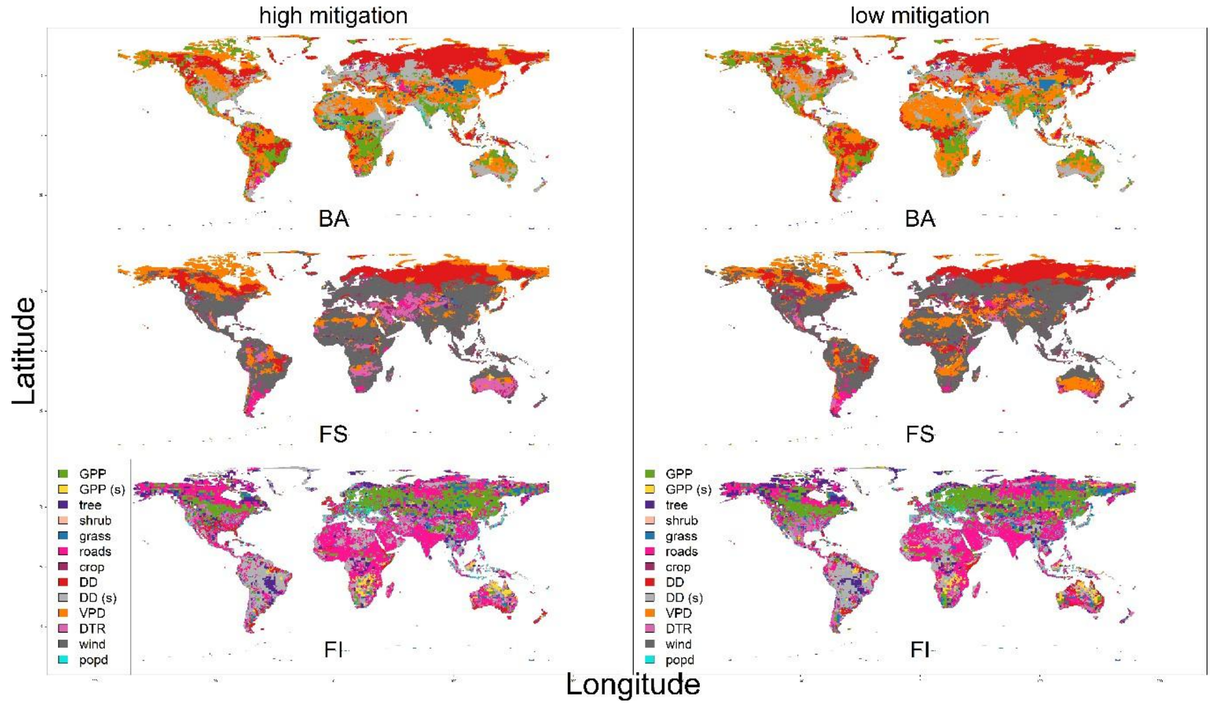

Supplementary Fig S10. Positive variable contribution for MIROC5. Map showing for each grid-cell which variable in the GLM model caused the positive contribution in the future projections for the high climate change mitigation (left) and low climate change mitigation (right) experiment for the MIROC5 climate model outputs for BA (first row), FS (second row) and FI (third row).

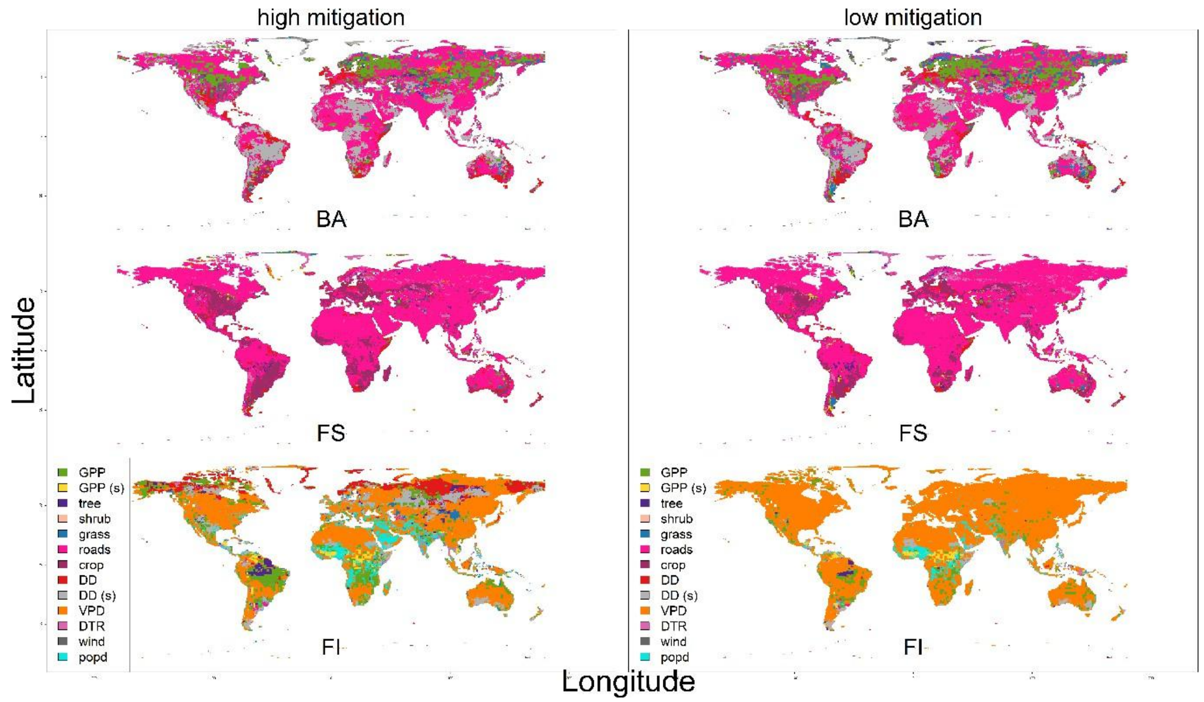

Supplementary Fig S11. Negative variable contribution for GFDL-ESM2M. Map showing for each grid-cell which variable in the GLM model caused the negative contribution in the future projections for the high climate change
mitigation (left) and low climate change mitigation (right) experiment for the GFDL-ESM2M climate model outputs for BA (first row), FS (second row) and FI (third row).

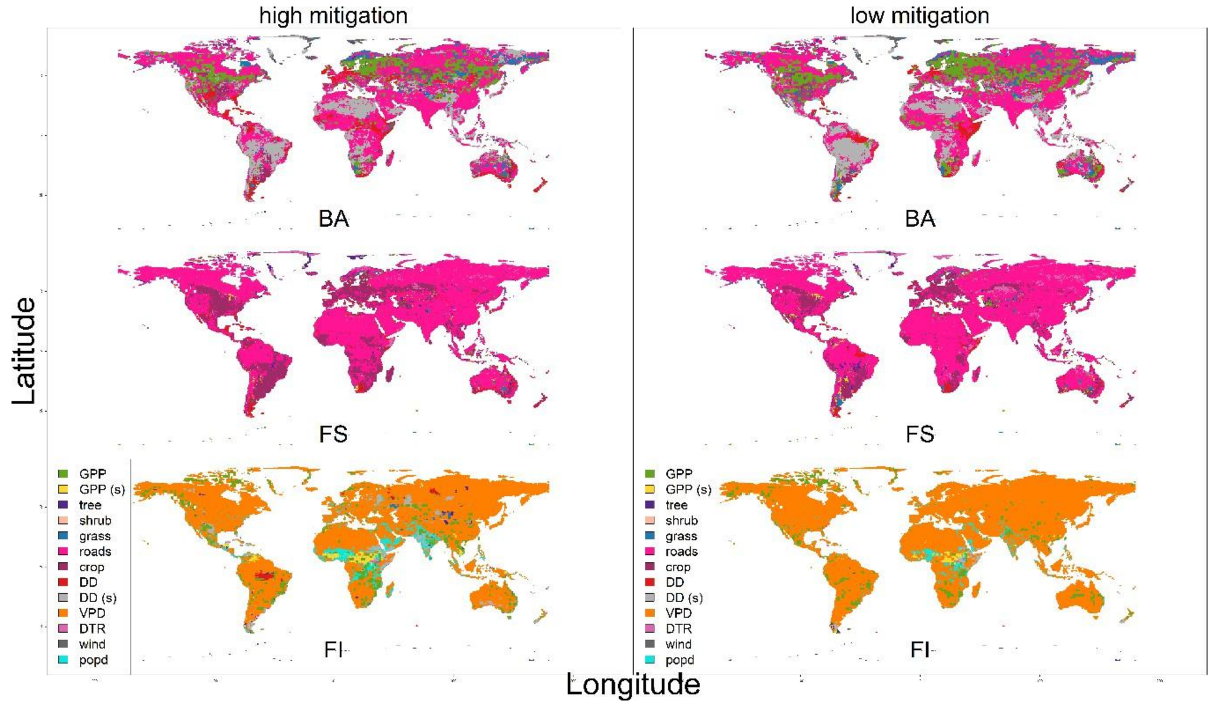

Supplementary Fig S12. Negative variable contribution for IPSL-CM5A-LR. Map showing for each grid-cell which variable in the GLM model caused the negative contribution in the future projections for the high climate change
mitigation (left) and low climate change mitigation (right) experiment for the IPSL-CM5A-LR climate model outputs for BA (first row), FS (second row) and FI (third row).

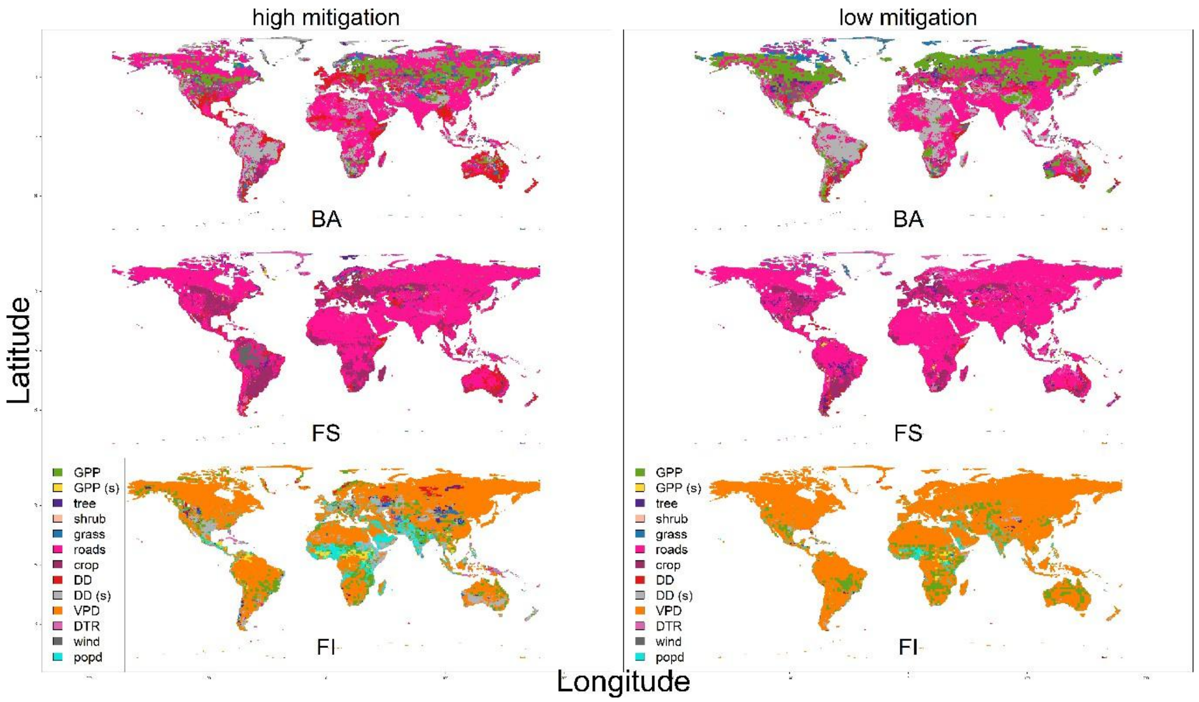

Supplementary Fig S13. Negative variable contribution for Had-GEM2-ES. Map showing for each grid-cell which variable in the GLM model caused the negative contribution in the future projections for the high climate change mitigation (left) and low climate change mitigation (right) experiment for the Had-GEM2-ES climate model outputs BA (first row), FS (second row) and FI (third row).

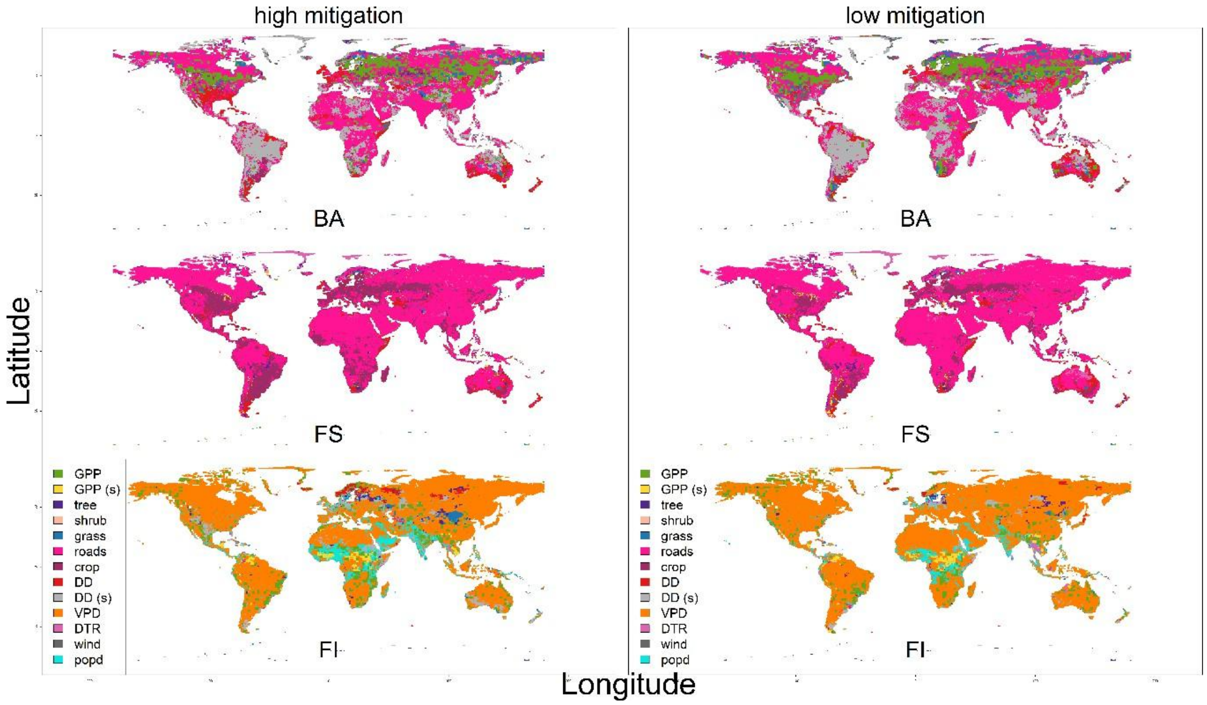

Supplementary Fig S14. Negative variable contribution for MIROC5. Map showing for each grid-cell which variable in the GLM model caused the negative contribution in the future projections for the high climate change mitigation (left) and low climate change mitigation (right) experiment for the MIROC5 climate model outputs for BA (first row), FS (second row) and FI (third row).

Supplementary Section 4: Climate only sensitivity experiments

All figures presented below are from the sensitivity experiments where only climate inputs were changed, while CO_2 levels (affecting vegetation) and human activity held constant under modern-day conditions. Under this sensitivity experiment, we still observe an overall decrease of global burnt area under the high climate change mitigation scenario, however this decrease is much less pronounced and dominated by sub-Saharan Africa, with increases simulated for example on the Indian subcontinent. Under the low climate change mitigation scenario, the simulations show increasing burnt area, but of a lesser magnitude than when all effects are included. The simulations show larger increases in fire size under both mitigation scenarios compared to when all effects are included and similar fire intensity changes.

Supplementary Table S5. Global for annual burnt area (BA), mean fire size (FS) and mean fire intensity (FI) values for the climate only experiments.

BA (km^2)	Modern-day (realistic)	High climate change mitigation	Percentage change	Low climate change mitigation	Percentage change
GFDL-ESM2M	4,059,433	3,493,071	-13.95	4,396,259	8.30
IPSL-CM5A-LR	4,059,433	4,436,700	9.30	4,359,014	7.38
HadGEM2-ES	4,059,433	3,748,743	-7.65	5,102,338	25.69
MIROC5	4,059,433	3,733,911	-8.02	3,360,245	-17.22
FS (km^2)	Modern-day (realistic)	High climate change mitigation	Percentage change	Low climate change mitigation	Percentage change
GFDL-ESM2M	2.84	3.66	29.04	3.93	38.53
IPSL-CM5A-LR	2.84	3.95	39.19	3.95	39.19
HadGEM2-ES	2.84	4.04	42.61	3.65	28.79
MIROC5	2.84	4.00	43.18	3.95	39.17
FI ($W.km^{-1}$)	Modern-day (realistic)	High climate change mitigation	Percentage change	Low climate change mitigation	Percentage change
GFDL-ESM2M	26.42	27.41	3.73	26.18	-0.94
IPSL-CM5A-LR	26.42	27.03	2.31	25.98	-1.67
HadGEM2-ES	26.42	27.76	5.06	24.03	-9.06
MIROC5	26.42	27.66	4.67	27.28	3.24

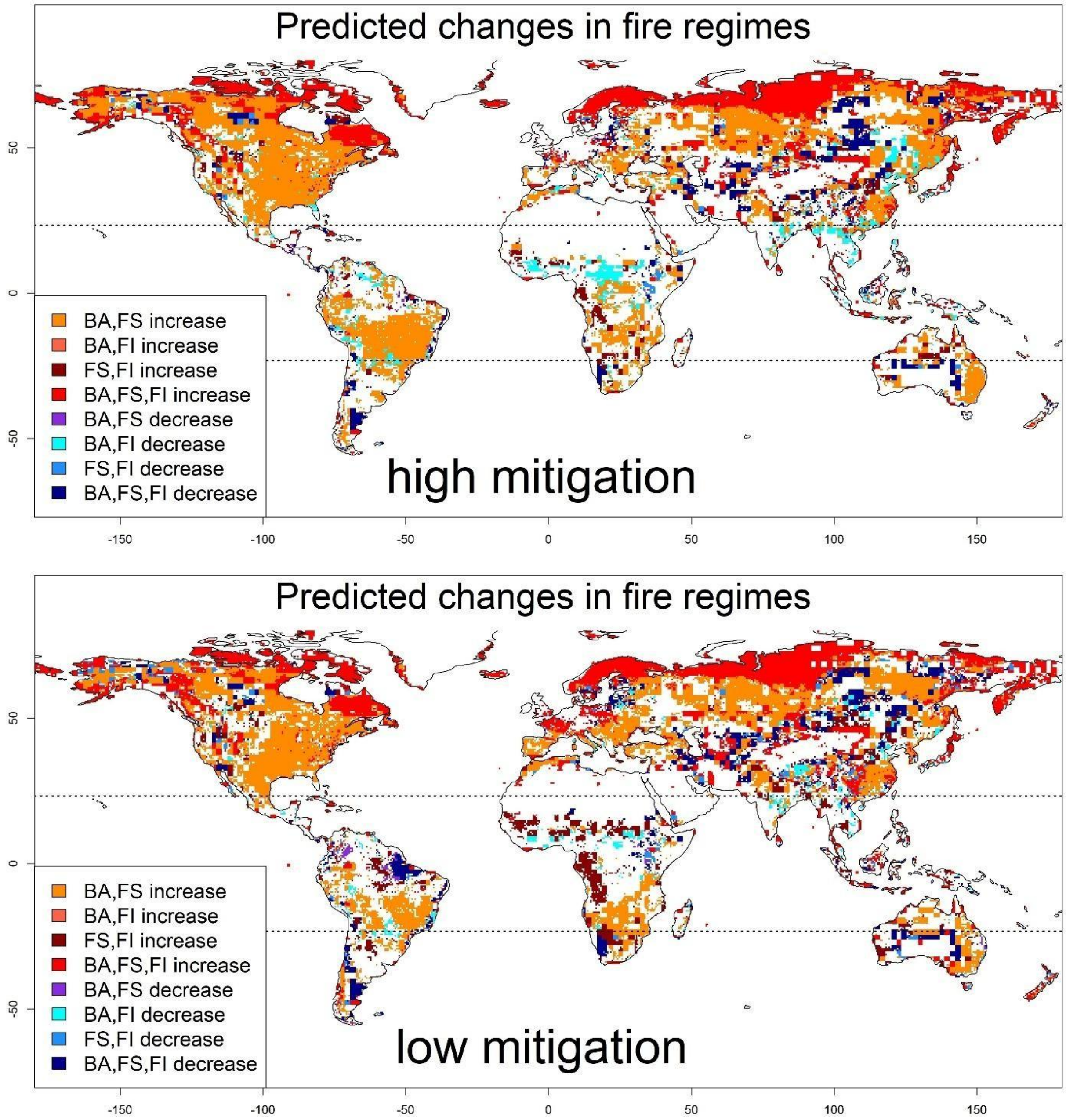

Supplementary Fig S15. Change in fire regimes by 2100 (climate only). Regions of change in burnt area (BA), fire size (FS) and fire intensity (FI) under the high climate change mitigation scenario (left) and the low climate change mitigation scenario (right) where CO_2 levels and human activity were held constant. Regions where all three properties increase are shown in red, regions where all three properties decrease are shown in dark blue. Base maps are produced using rnaturalearth.

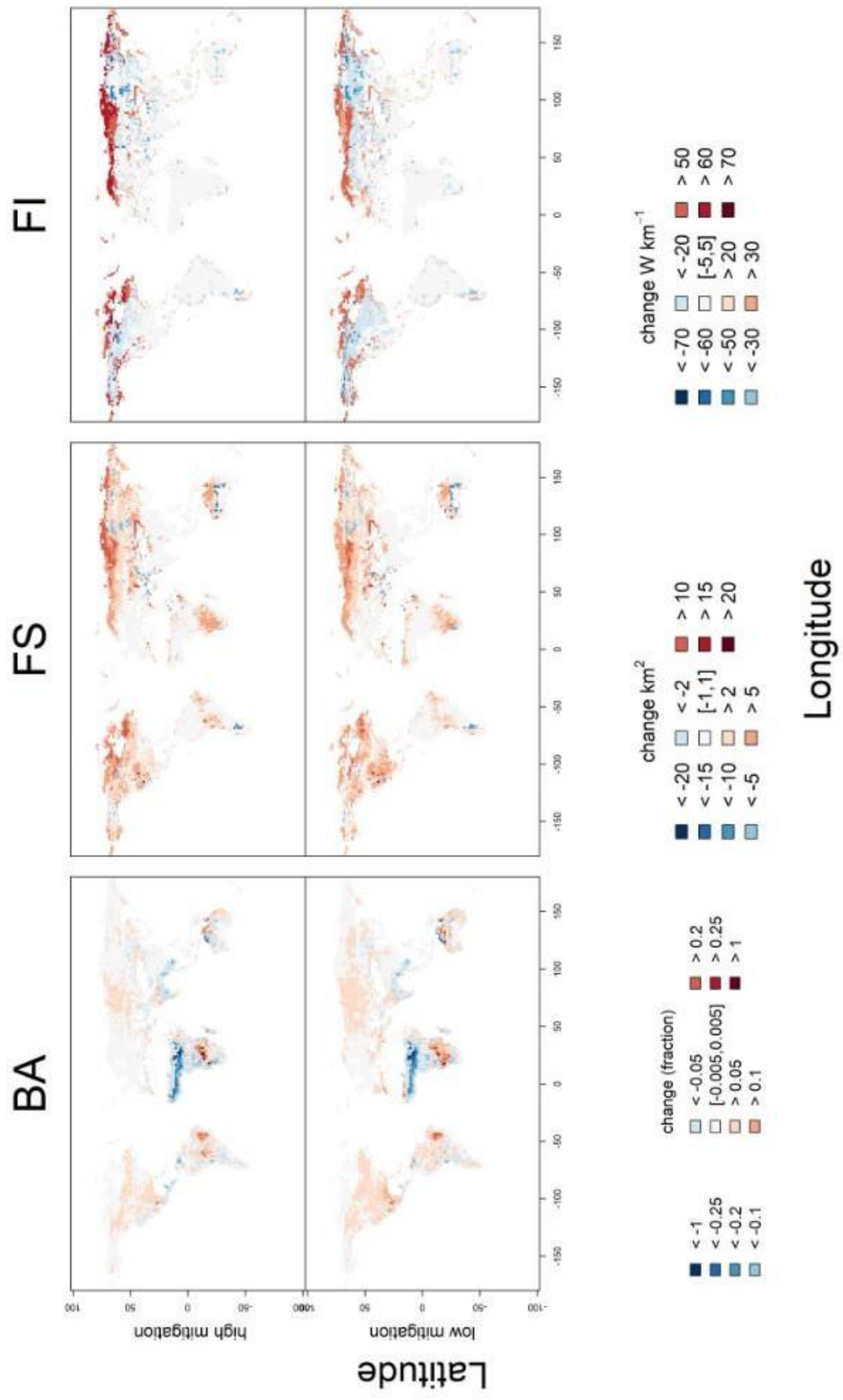

Supplementary Fig S16. Fire properties anomalies (climate only). Anomalies for burnt area (BA; first column), fire size (FS; second column) and fire intensity (FI; third column) between the modern-day experiment and the high climate change mitigation experiment (top panel) and low climate change mitigation experiment (bottom panel) for the mean of the experiments driven by the outputs of all four climate models in which CO_2 levels are held constant at 395ppm and human activity is held constant at the 2010-2015 baseline. Red represents an increase in the fire property by the end of the century and blue represents a decrease.

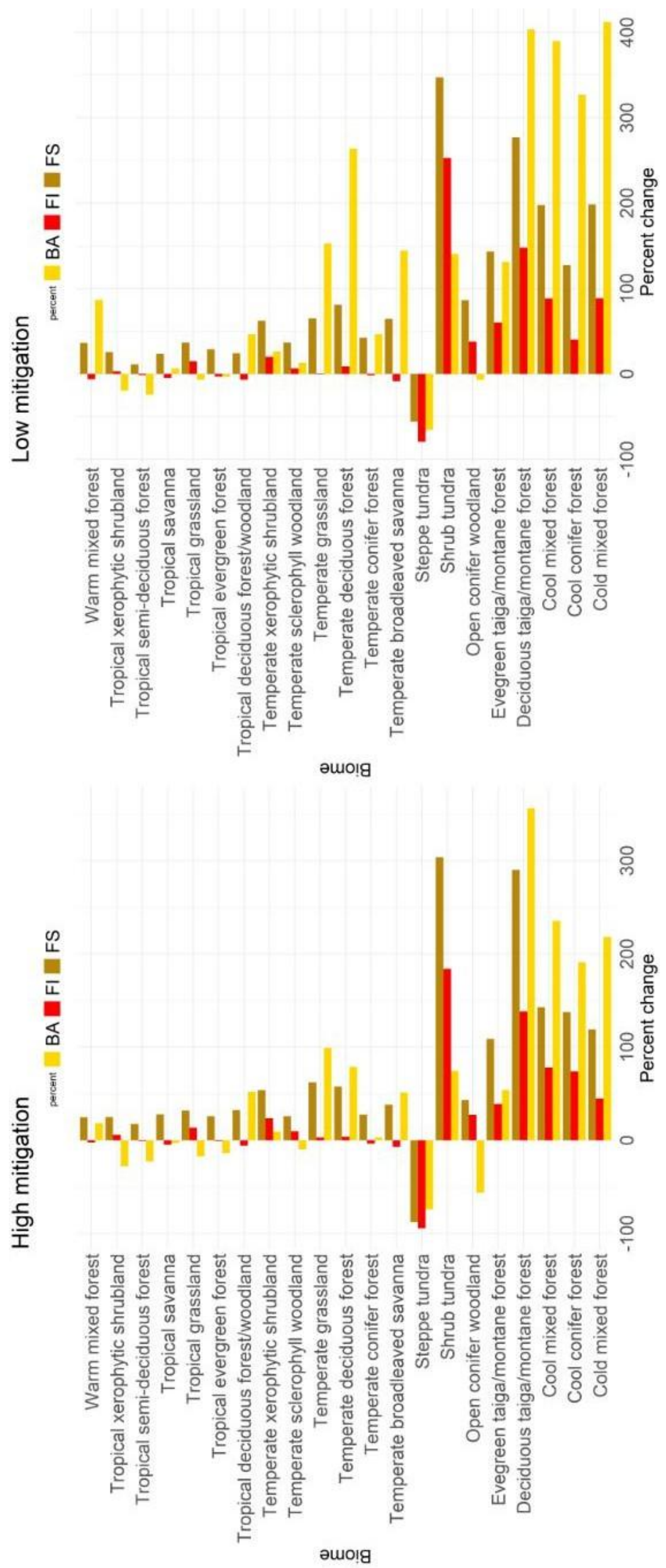

Supplementary Fig S17. Biome breakdown of predicted change (climate only). Percentage change for all three fire properties between realistic modern-day conditions and the climate only future experiments broken down by biome type. The mean is taken across all four climate-model experiments for each scenario.

Supplementary Section 5: CO_2 only sensitivity experiments

All figures presented below are from the sensitivity experiments where only changes were allowed in CO_2 levels (affecting vegetation; 424ppm under RCP2.6 and 651ppm under RCP6.0) and climate human activity held constant under modern-day conditions. Under this sensitivity analysis, burnt area increases under both mitigation scenarios, and the increase under the low climate change mitigation scenario is much more pronounced than when all effects are included. This highlights the effect of increased CO_2 levels on increasing burning through its effect on vegetation. Both fire size and fire intensity show very small global decreases under this experiment, which are driven by simulated decreases across southern Africa and North America. This decrease in North America is not simulated in any other experiment.

Supplementary Table S6. Global annual burnt area (BA), mean fire size (FS) and mean fire intensity (FI) values for the CO_2 only experiments.

BA (km^2)	Modern-day (realistic)	High climate change mitigation	Percentage change	Low climate change mitigation	Percentage change
	4,059,433	4,466,470	10.03	6,997,835	72.89
FS (km^2)	Modern-day (realistic)	High climate change mitigation	Percentage change	Low climate change mitigation	Percentage change
	2.84	2.82	-0.66	2.80	-1.38
FI ($W.km^{-1}$)	Modern-day (realistic)	High climate change mitigation	Percentage change	Low climate change mitigation	Percentage change
	26.42	26.27	-0.58	25.43	-3.76

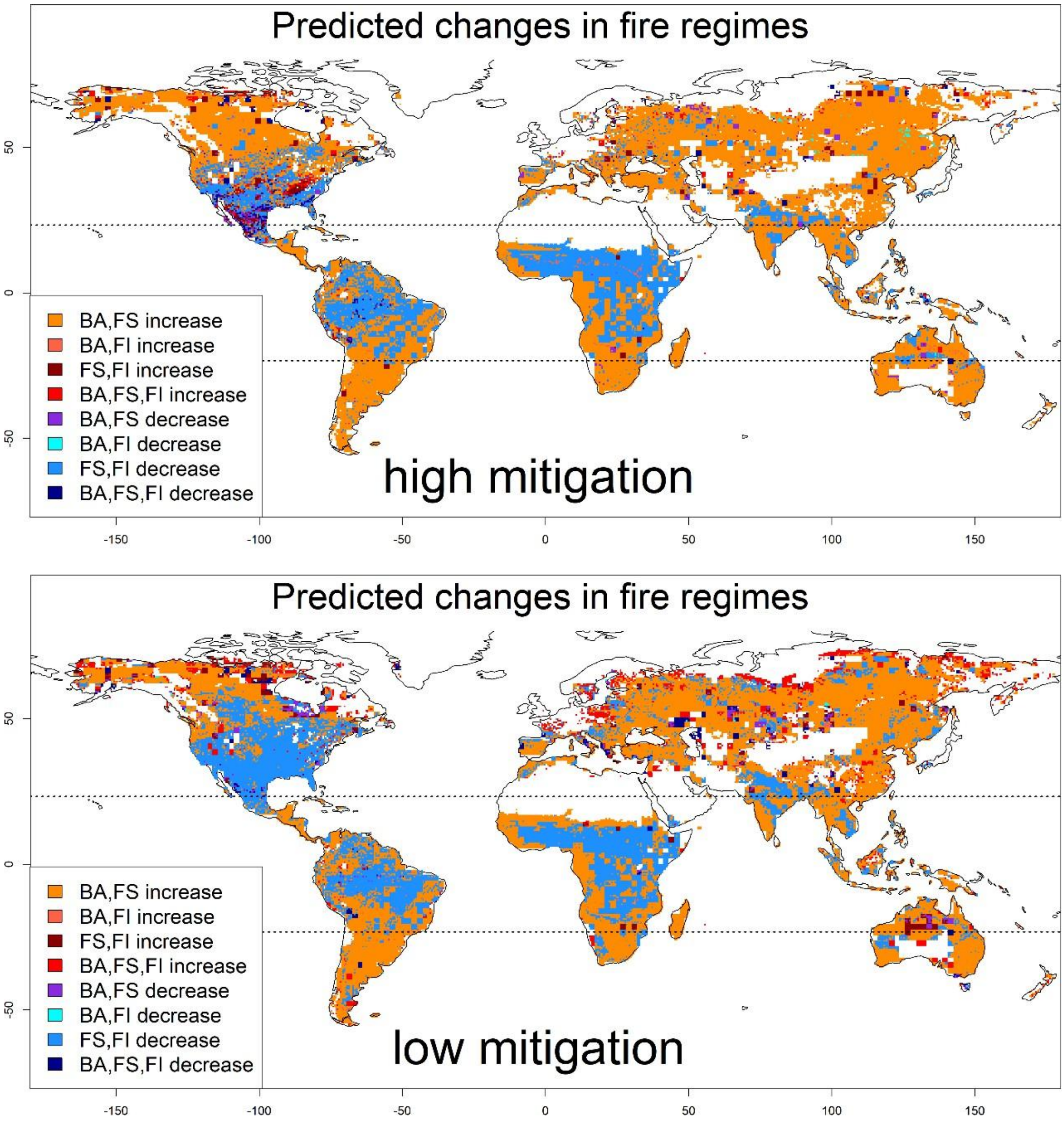

Supplementary Fig S18. Change in fire regimes by 2100 (CO_2 only). Regions of change in burnt area (BA), fire size (FS) and fire intensity (FI) under the high climate change mitigation scenario (left) and the low climate change mitigation scenario (right) where climate and human activity were held constant. Regions where all three properties increase are shown in red, regions where all three properties decrease are shown in dark blue. Base maps are produced using rnaturalearth.

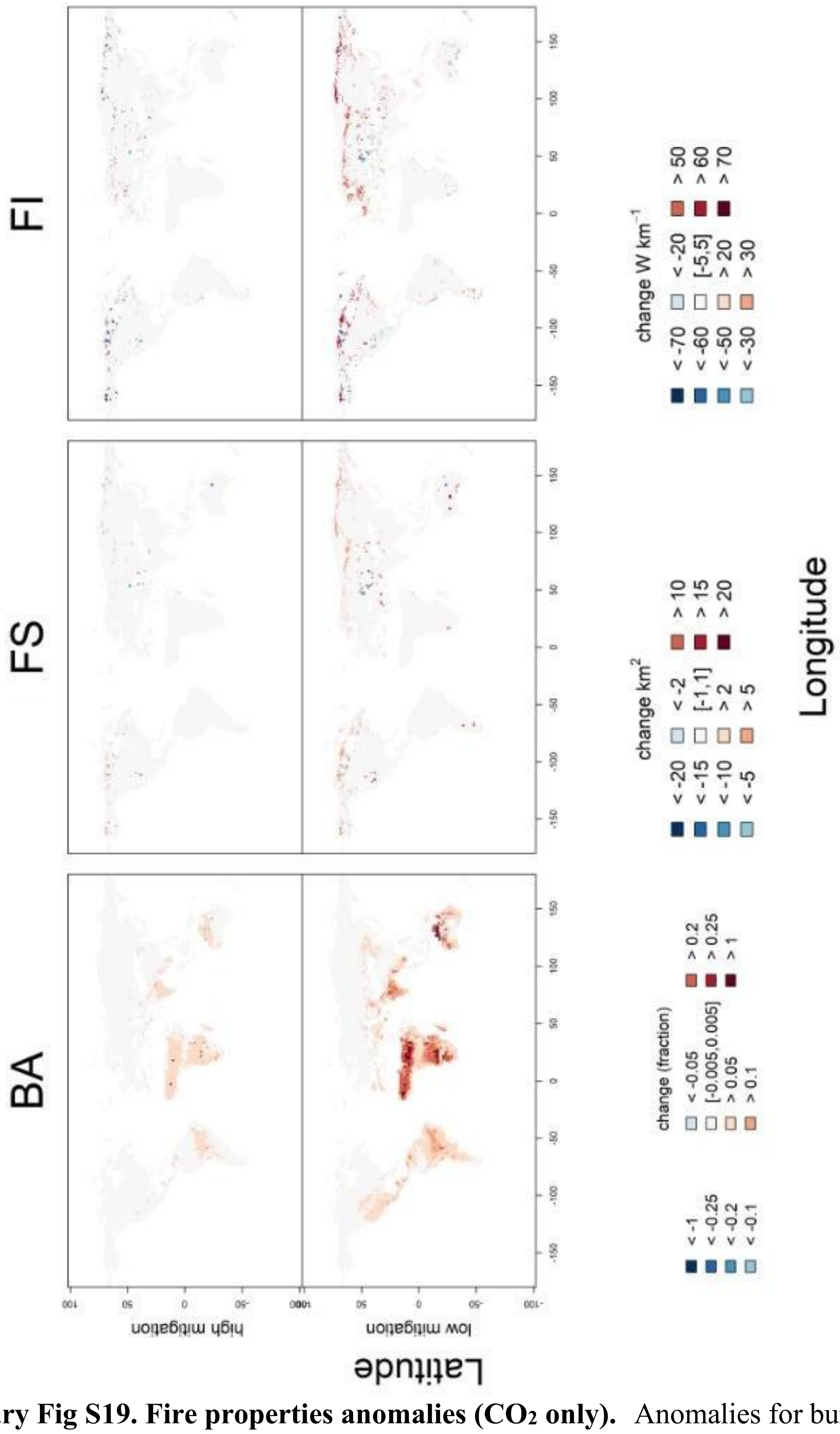

Supplementary Fig S19. Fire properties anomalies (CO_2 only). Anomalies for burnt area (BA; first column), fire size (FS; second column) and fire intensity (FI; third column) between the modern-day experiment and the high climate change mitigation experiment (top panel) and low climate change mitigation experiment (bottom panel) for the mean of the experiments driven by the outputs of all four climate models in which CO_2 levels are 424ppm and 651ppm respectively and climate human activity are held constant at the 2010-2015 baseline. Red represents an increase in the fire property by the end of the century and blue represents a decrease.

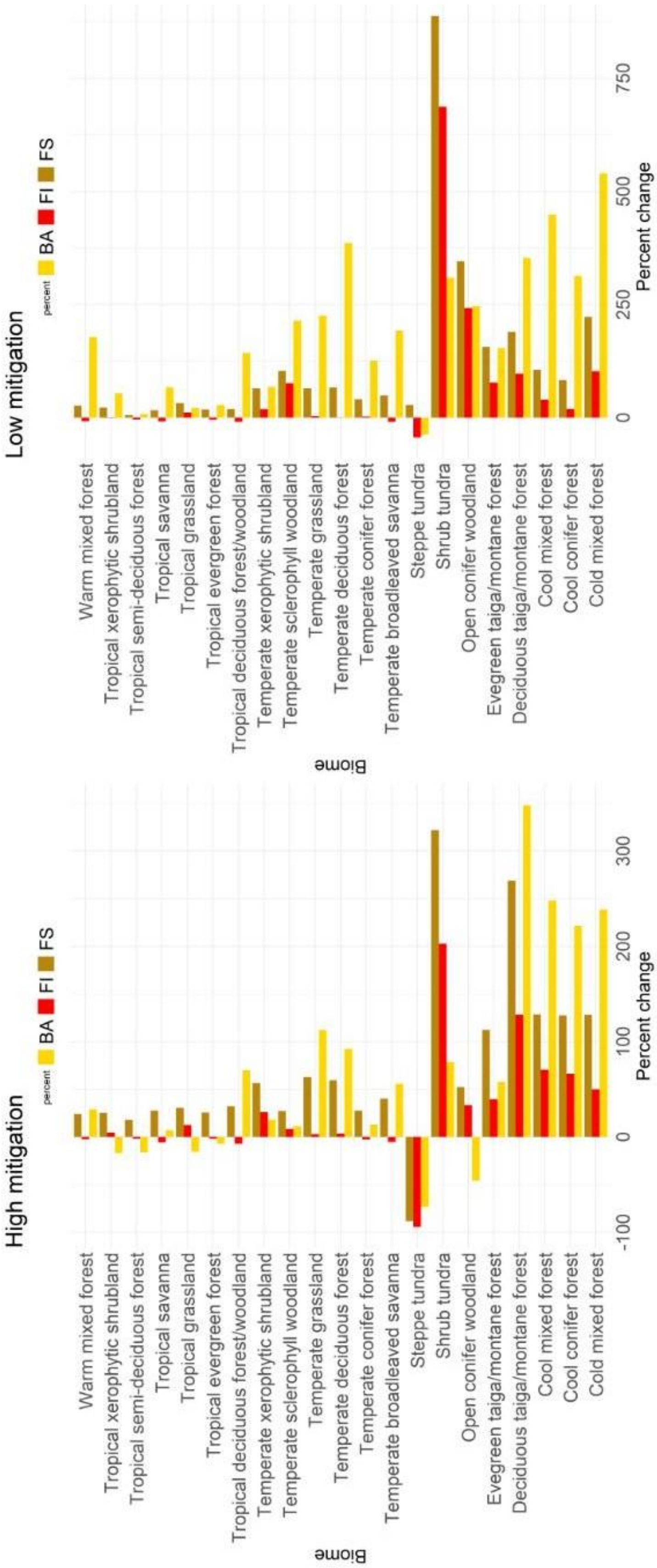

Supplementary Fig S20. Biome breakdown of predicted change (CO_2 only). Percentage change for all three fire properties between realistic modern-day conditions and the CO_2 only future experiments broken down by biome type. The mean is taken across all four climate-model experiments for each scenario.

Supplementary Section 6: Climate and CO_2 only sensitivity experiments

All figures presented below are from the sensitivity experiments where climate inputs and CO_2 levels were changed, while human activity was held constant under modern-day conditions. Under this experiment, we see increases under both mitigation scenarios. Under the high climate change mitigation scenario, the increase due to CO_2 levels (seen in the previous CO_2 levels experiment) are dampened by climate effect (which is globally decreasing under the climate only experiment). However, under low climate change mitigation, both effects are positive and taken together, amplify each other, leading to large increases in burnt area. Large increases in burnt area are shown in southern Africa and in Southern Brazil under both mitigation scenarios. There is an increase in fire size under this experiment compared to the experiment where all effects are included but similar fire intensity to the full experiment.

Supplementary Table S7. Global annual burnt area (BA), mean fire size (FS) and mean fire intensity (FI) values for the climate and CO_2 only experiments.

BA (km^2)	Modern-day (realistic)	High climate change mitigation	Percentage change	Low climate change mitigation	Percentage change
GFDL-ESM2M	4,059,433	3856381	-5.001989	7692990	89.50898
IPSL-CM5A-LR	4,059,433	4876644	20.1316	8063178	98.62818
HadGEM2-ES	4,059,433	4156526	2.391791	8141939	100.5684
MIROC5	4,059,433	4123215	1.571191	5949635	46.56319

FS (km^2)	Modern-day (realistic)	High climate change mitigation	Percentage change	Low climate change mitigation	Percentage change
GFDL-ESM2M	2.84	3.66	28.94	3.96	39.74
IPSL-CM5A-LR	2.84	3.95	39.6	3.91	37.95
HadGEM2-ES	2.84	4.03	42.35	3.65	28.75
MIROC5	2.84	4.068125	43.45373	3.912421	37.9636
FI ($W.km^{-1}$)	Modern-day (realistic)	High climate change mitigation	Percentage change	Low climate change mitigation	Percentage change
GFDL-ESM2M	26.42	27.22	3.02	25.01	-5.34
IPSL-CM5A-LR	26.42	26.86	1.64	24.53	-7.17
HadGEM2-ES	26.42	27.62	4.54	22.88	-13.40
MIROC5	26.42	27.61	4.48	25.98	-1.69

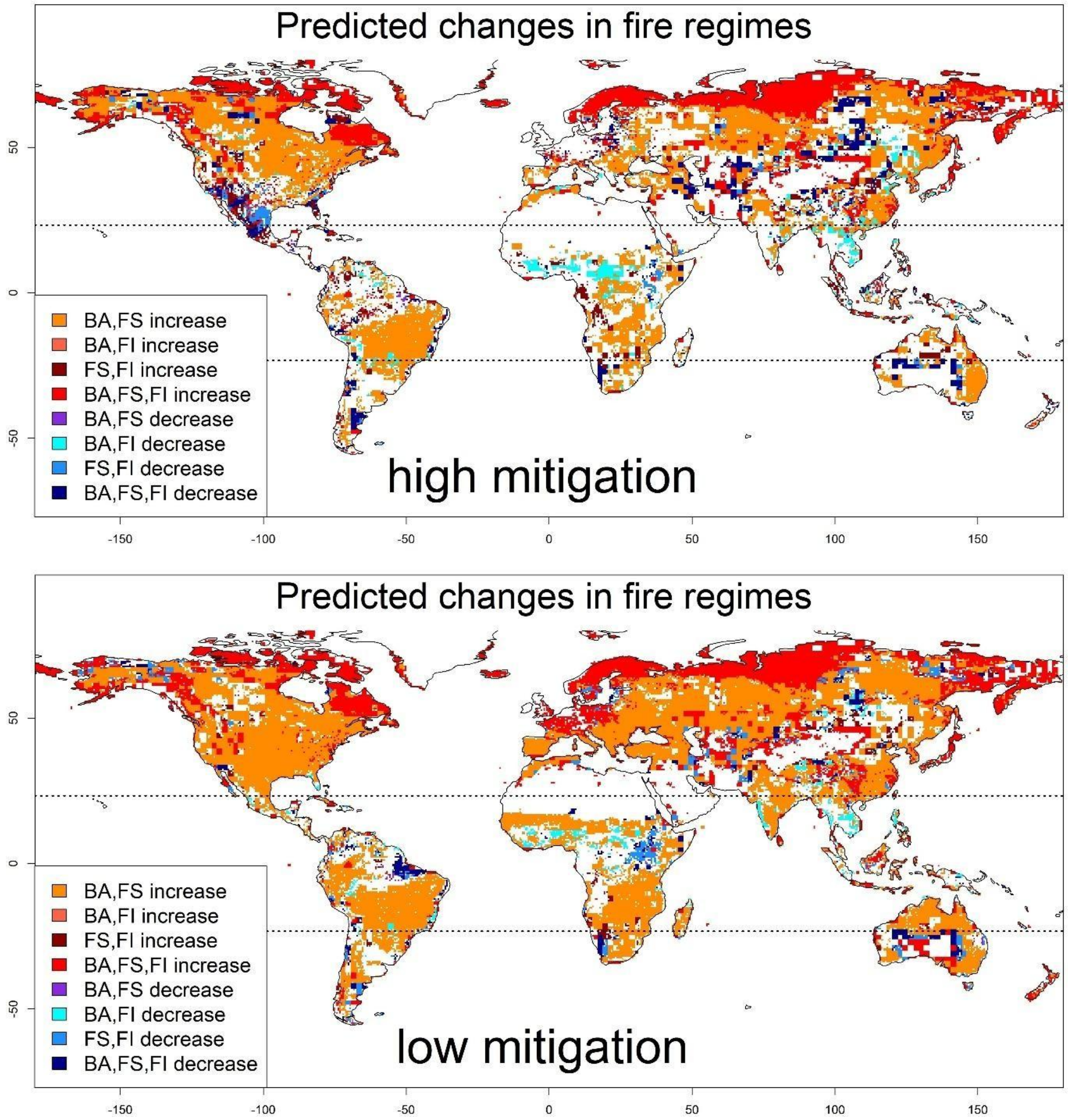

Supplementary Fig S21. Change in fire regimes by 2100 (CO_2 and climate only). Regions of change in burnt area (BA), fire size (FS) and fire intensity (FI) under the high climate change mitigation scenario (left) and the low climate change mitigation scenario (right) where human activity were held constant. Regions where all three properties increase are shown in red, regions where all three properties decrease are shown in dark blue. Base maps are produced using rnaturalearth.

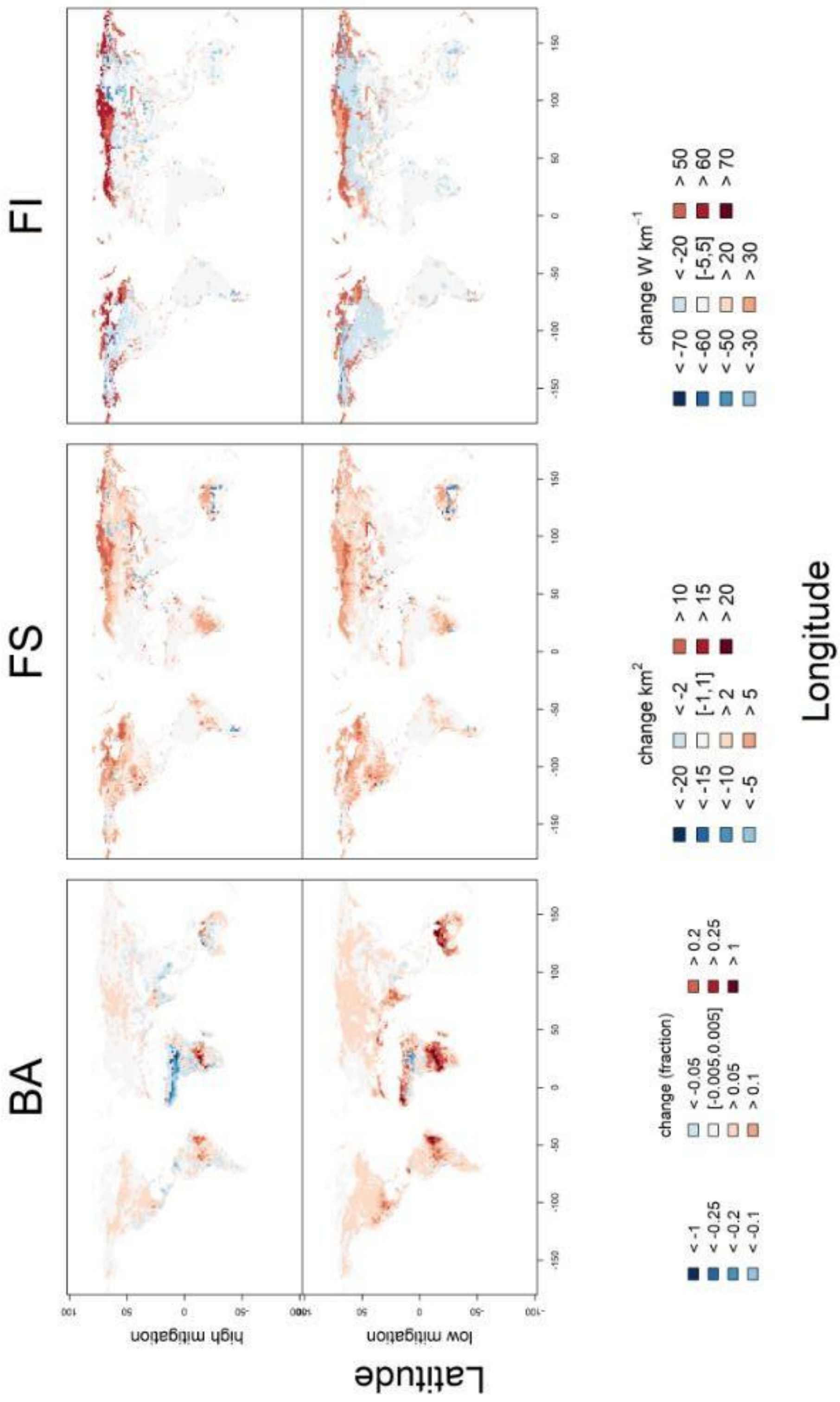

Supplementary Fig S22. Fire properties anomalies (CO_2 and climate only). Anomalies burnt area (BA; first column), fire size (FS; second column) and fire intensity (FI; third column) between the modern-day experiment and the high climate change mitigation experiment (top panel) and low climate change mitigation experiment (bottom panel) for the mean of the experiments driven by the outputs of all four climate models in which CO_2 levels are 424ppm and 651ppm respectively and human activity are held constant at the 2010-2015 baseline. Red represents an increase in the fire property by the end of the century, and blue represents a decrease.

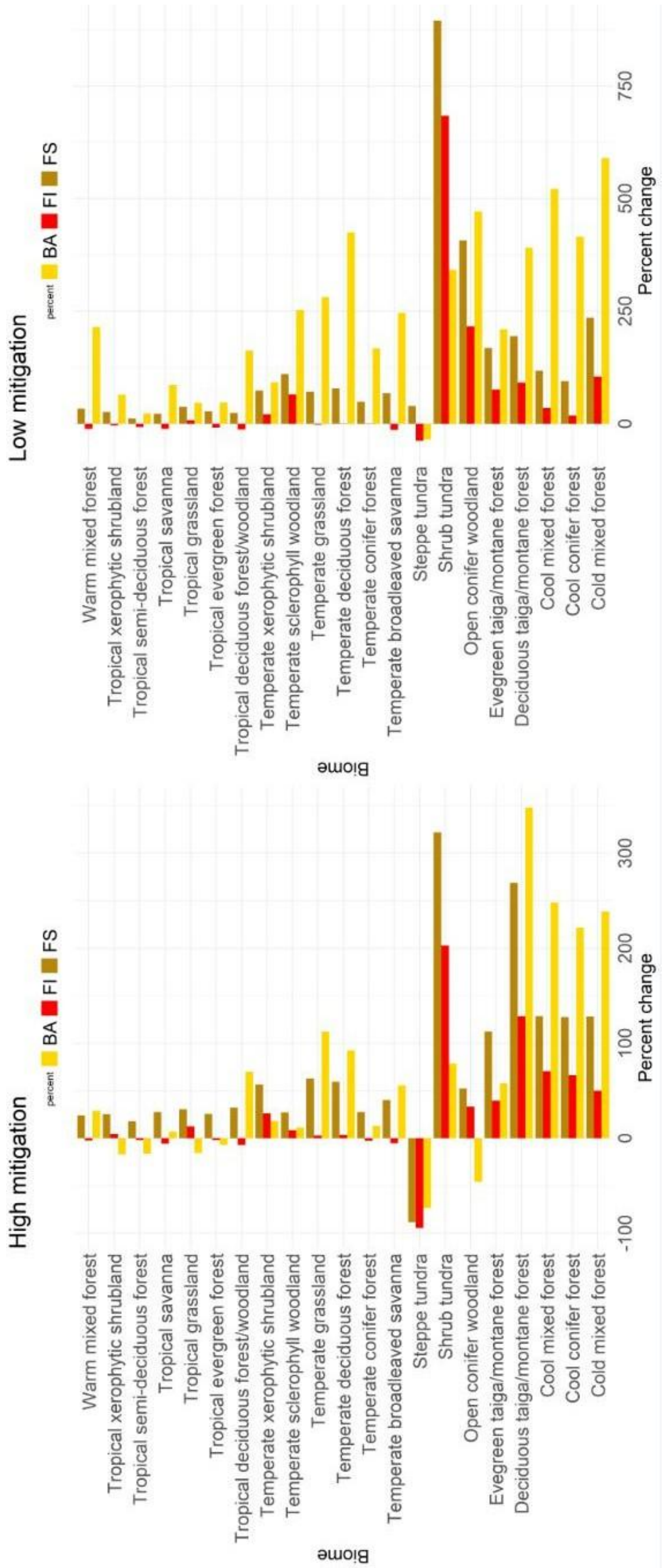

Supplementary Fig S23. Biome breakdown of predicted change (CO_2 and climate only). Percentage change for all three fire properties between realistic modern-day conditions and the climate and CO_2 only future experiments broken down by biome type. The mean is taken across all four climate-model experiments for each scenario.

Supplementary Section 7: Human activity only sensitivity experiments

All figures presented below are from the sensitivity experiments where human activity (SSP2 scenario) was changed, while climate inputs and CO_2 levels (affecting vegetation) are held constant under modern-day conditions. Under this experiment we see decreasing burnt area under both mitigation scenarios (of similar magnitude to the full, low climate change mitigation experiment for both human only experiments). We also see decreasing fire size, with slightly larger decreases under the low climate change mitigation scenario than under the high climate change mitigation scenario. We see a very slight (less than 1%) increase in fire intensity in both mitigation scenarios. Decreases are simulated everywhere except for south-east Asia where there is an increase in burnt area, fire size and fire intensity under both scenarios. This is also the casse for regions in central Europe and North America.

Supplementary Table S8. Global annual burnt area (BA), mean fire size (FS) and mean fire intensity (FI) values for human only experiments.

BA (km^2)	Modern-day (realistic)	High climate change mitigation	Percentage change	Low climate change mitigation	Percentage change
GFDL-ESM2M	4,059,433	3245393	-20.05306	321961	-20.69926
IPSL-CM5A-LR	4,059,433	3247321	20.00556	3215363	-20.79282
HadGEM2-ES	4,059,433	3242333	-20.12844	3206252	-21.01724
MIROC5	4,059,433	3246905	-20.01579	3228145	-20.47794
FS (km^2)	Modern-day (realistic)	High climate change mitigation	Percentage change	Low climate change mitigation	Percentage change
GFDL-ESM2M	2.84	2.67	-5.84	2.62	-7.69
IPSL-CM5A-LR	2.84	2.66	-6.10	2.61	-7.80
HadGEM2-ES	2.84	2.66	-6.02	2.61	-7.80
MIROC5	2.84	2.66	-6.22	2.62	-7.48
FI ($W.km^{-1}$)	Modern-day (realistic)	High climate change mitigation	Percentage change	Low climate change mitigation	Percentage change
GFDL-ESM2M	26.42	26.55	0.46	26.55	0.46
IPSL-CM5A-LR	26.42	26.55	0.46	26.55	0.47
HadGEM2-ES	26.42	26.55	0.47	26.55	0.47
MIROC5	26.42	26.55	0.46	26.55	0.46

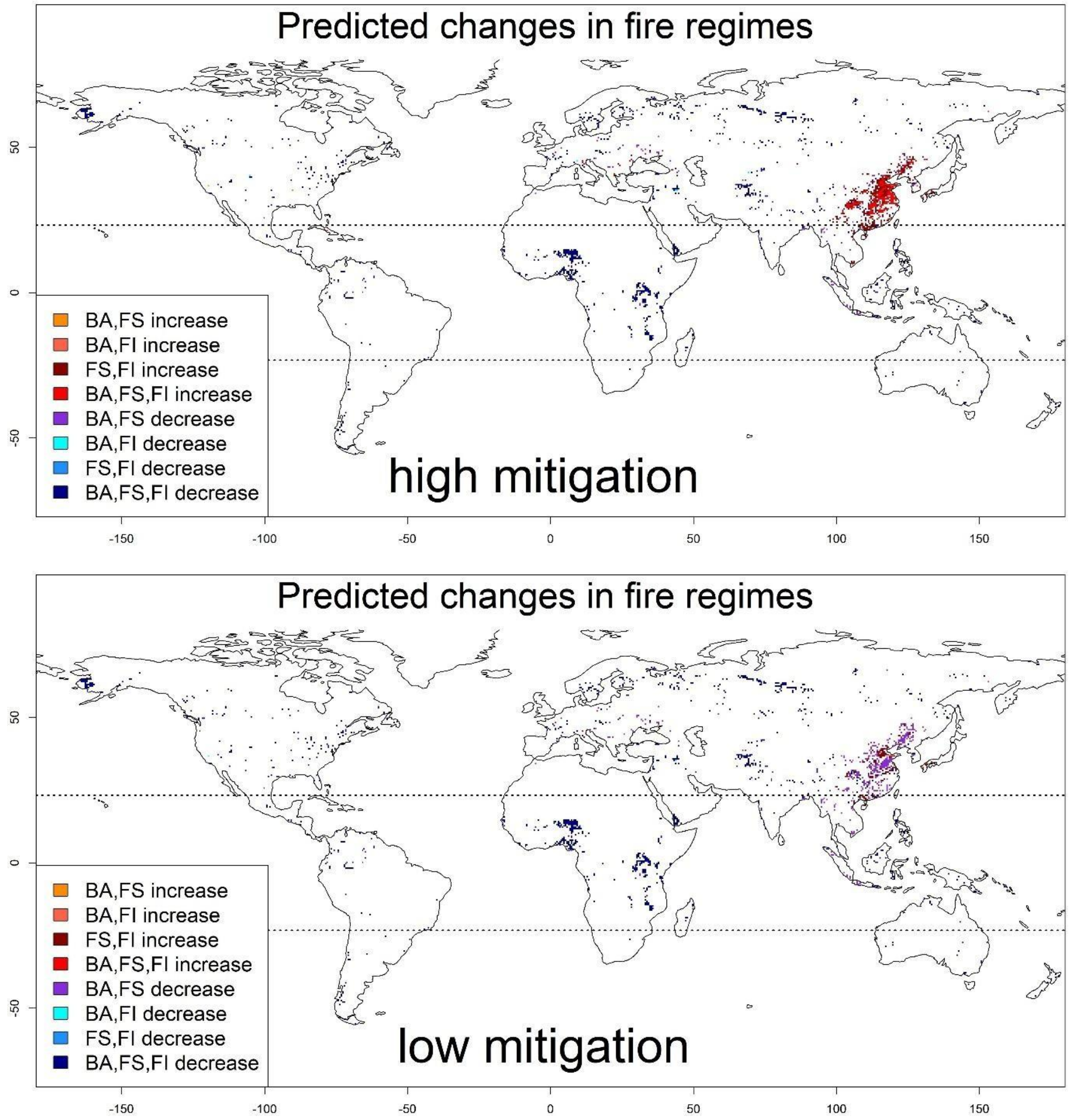

Supplementary Fig S24. Change in fire regimes by 2100 (CO_2 and climate only). Regions of change in burnt area (BA), fire size (FS) and fire intensity (FI) under the high climate change mitigation scenario (left) and the low climate change mitigation scenario (right) where climate and CO2 were held constant. Regions where all three properties increase are shown in red, regions where all three properties decrease are shown in dark blue. Base maps are produced using rnaturalearth.

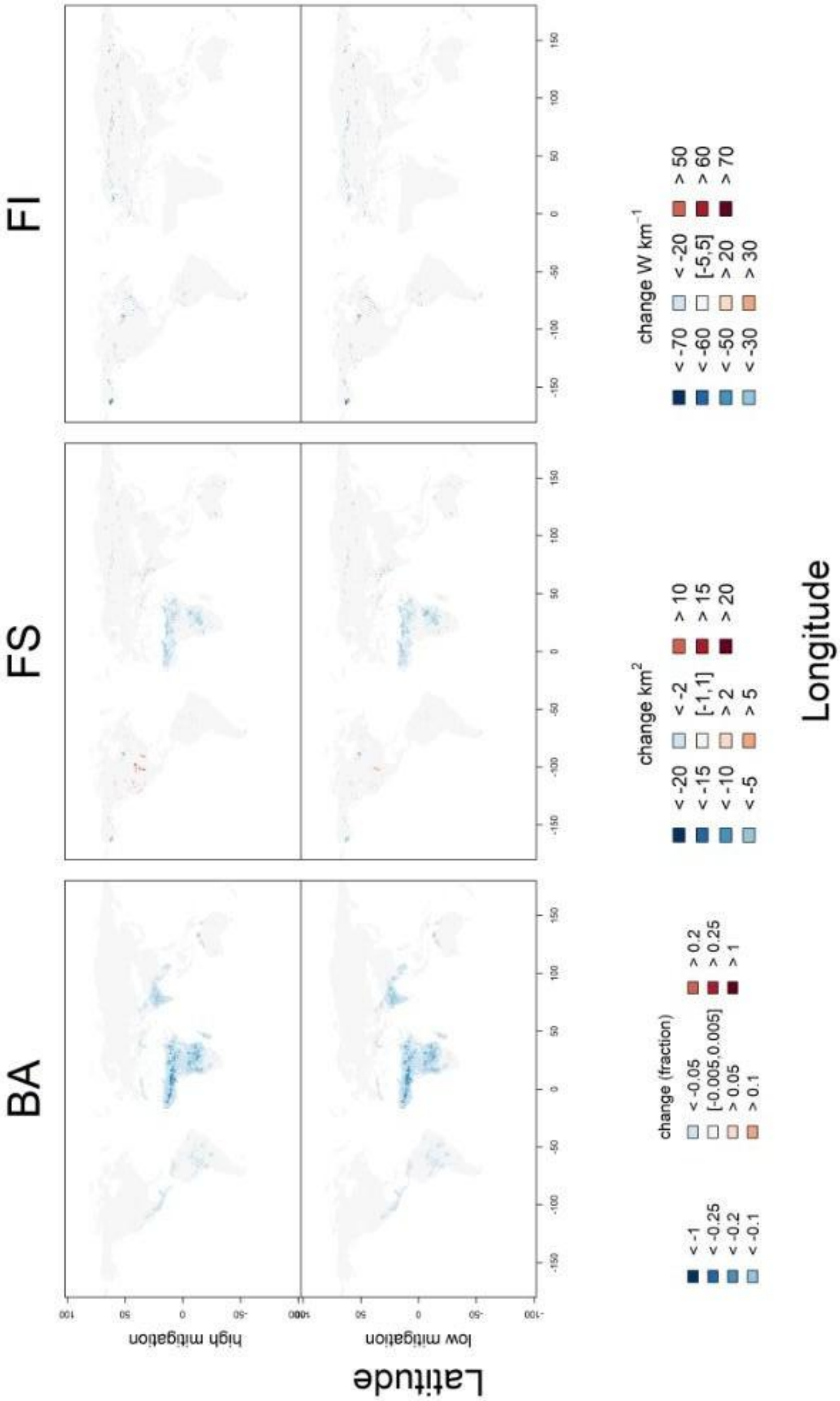

Supplementary Fig S25. Fire properties anomalies (human activity only). Anomalies burnt area (BA; first column), fire size (FS; second column) and fire intensity (FI; third column) between the modern-day experiment and the high climate change mitigation experiment (top panel) and low climate change mitigation experiment (bottom panel) for the mean of the experiments driven by the outputs of all four climate models in which only human activity changes (SSP2 scenario) and in which climate and CO_2 levels are held constant at the 2010-2015 baseline. Red represents an increase in the fire property by the end of the century and blue represents a decrease.

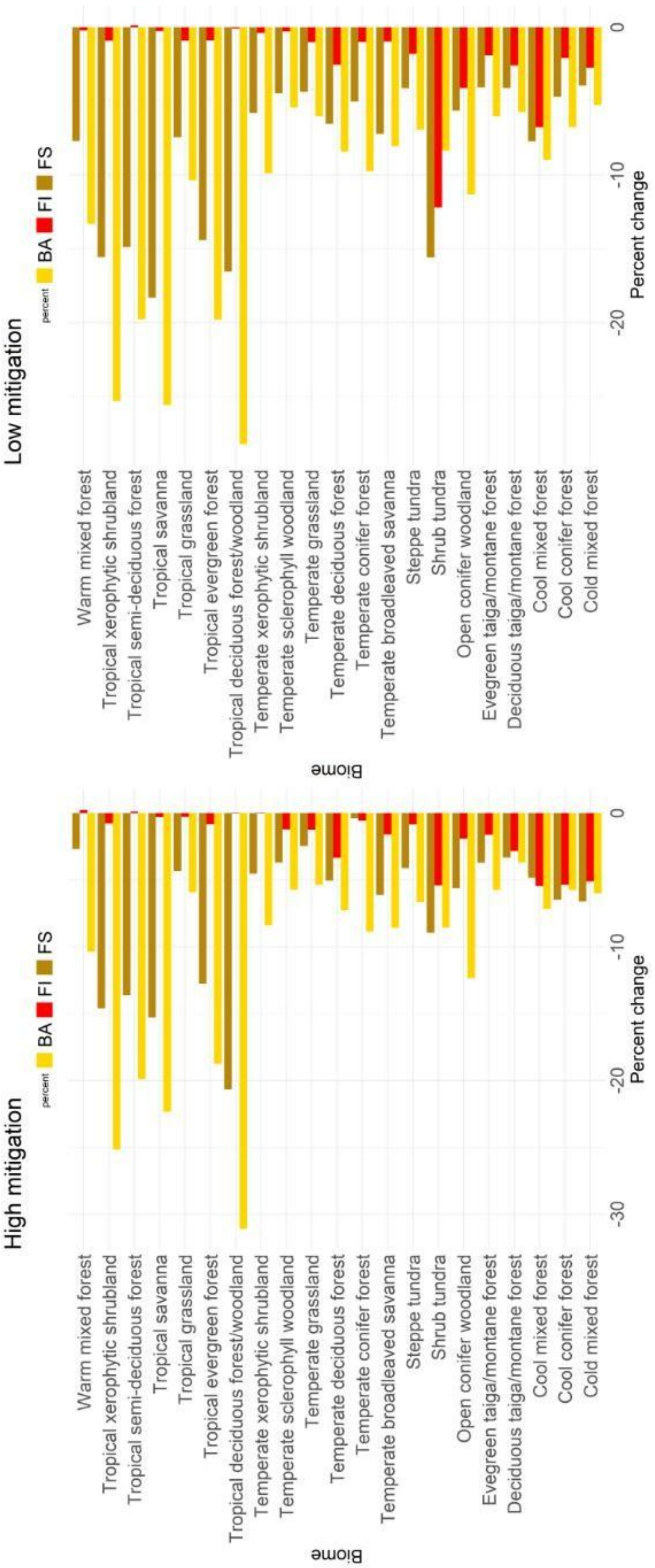

Supplementary Fig S26. Biome breakdown of predicted change (Human activity only). Percentage change for all three fire properties between realistic modern-day conditions and human activity only future experiments broken down by biome type. The mean is taken across all four climate-model experiments for each scenario.

Supplementary Section 8: Change by the end of century in the climate, vegetation and human activity under the high and low climate change mitigation scenarios

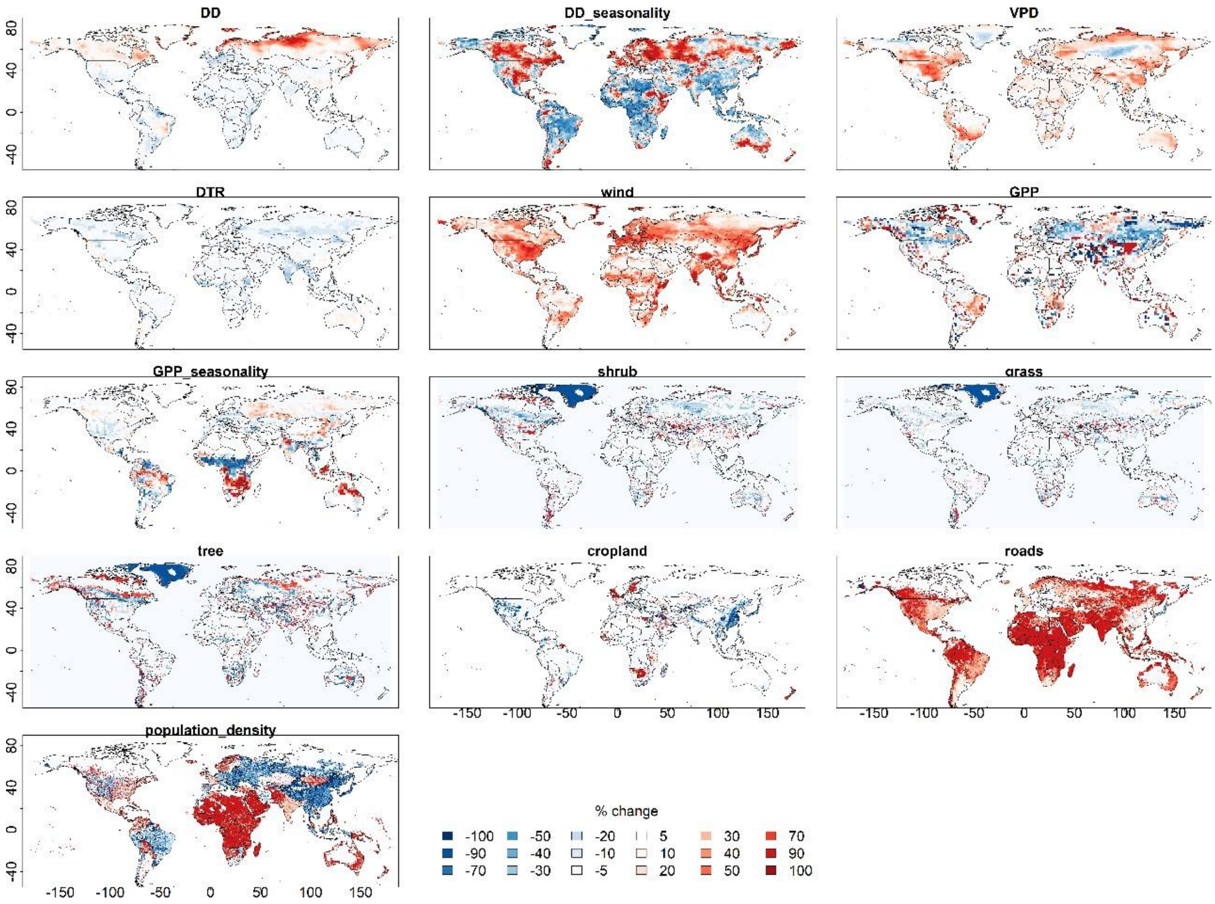

Supplementary Fig S27. **Percentage change in input variables for GFDL-ESM2M (high climate change mitigation).** Percentage change in all predictors between the modern-day and the end of century high climate change mitigation scenarios for GFDL-ESM2M climate model outputs.

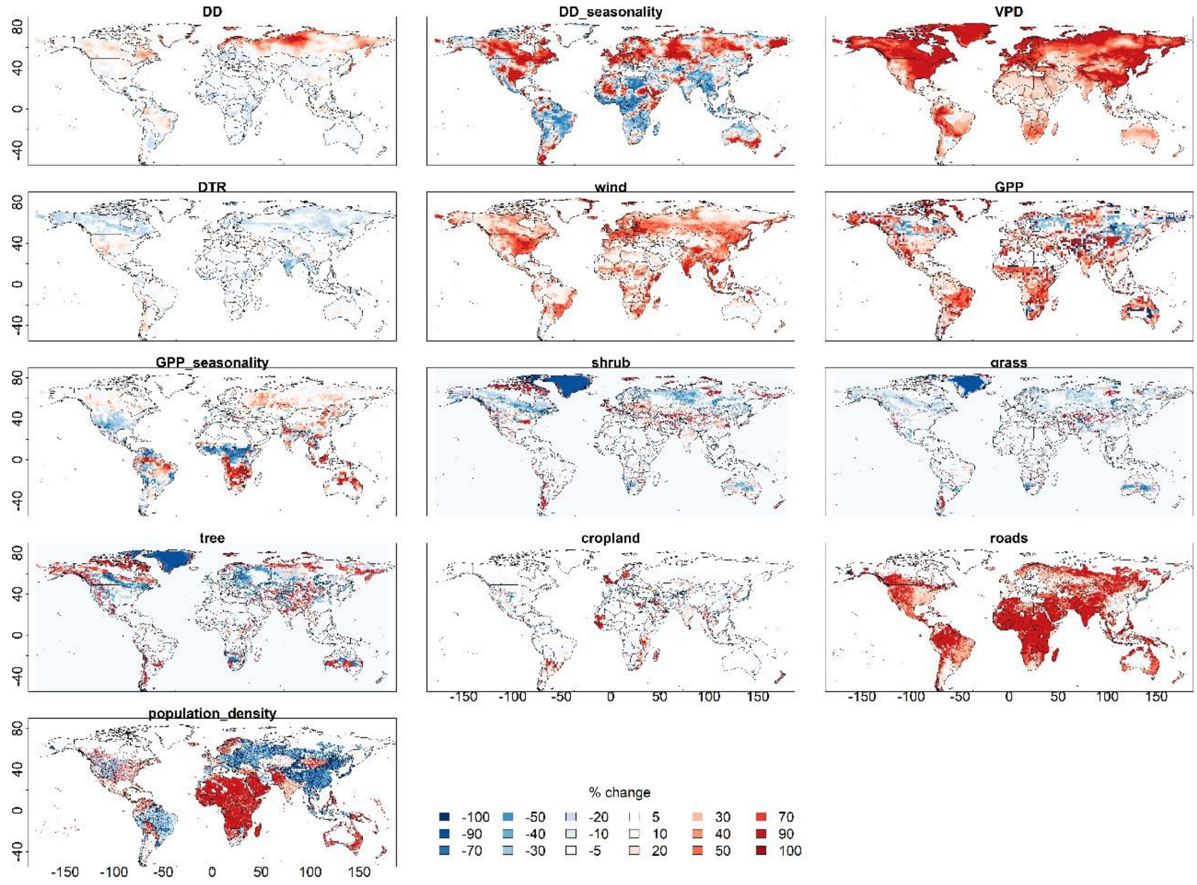

Supplementary Fig S28. Percentage change in input variables for GFDL-ESM2M (low climate change mitigation). Percentage change in all predictors between the modern-day and the end of century low climate change mitigation scenarios for GFDL-ESM2M climate model outputs.

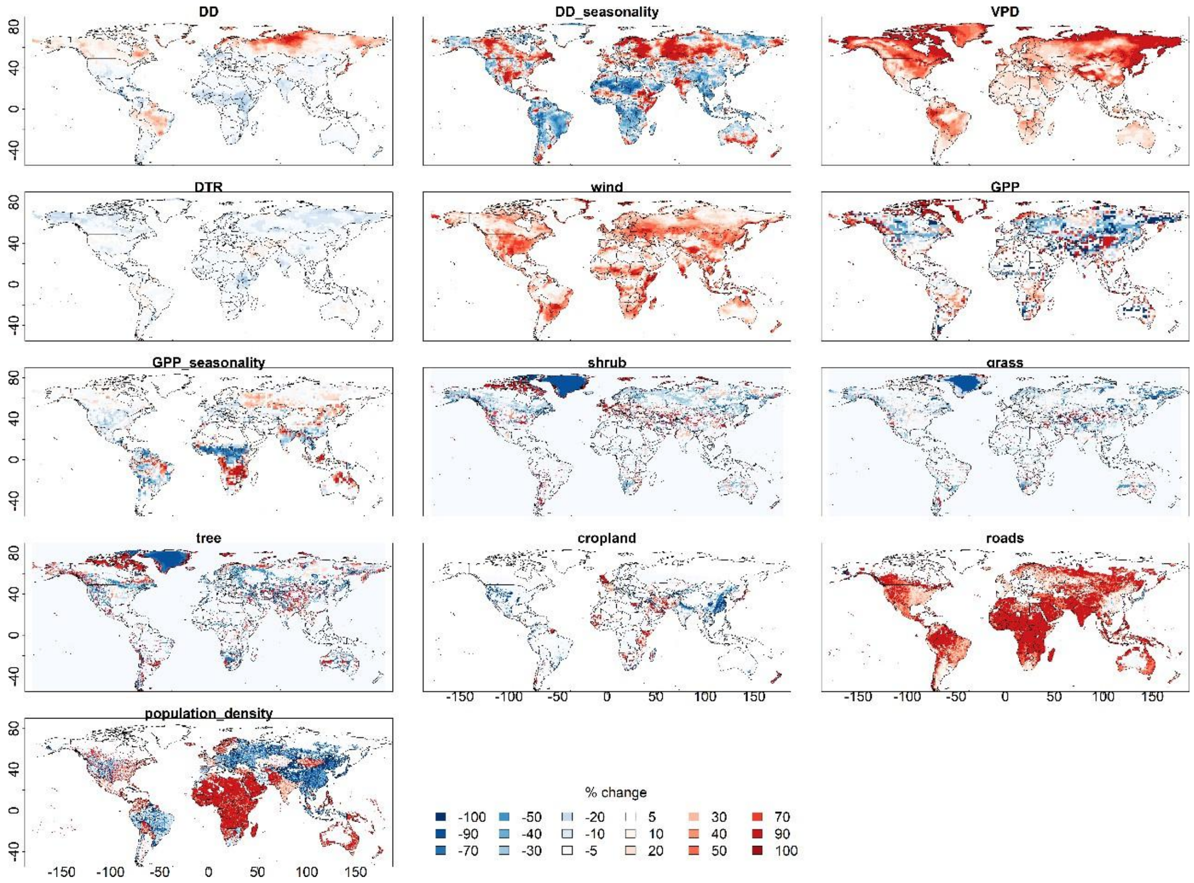

Supplementary Fig S29. **Percentage change in input variables for IPSL-CM5A-LR (high climate change mitigation).** Percentage change in all predictors between the modern-day and the end of century high climate change mitigation scenarios for IPSL-CM5A-LR climate model outputs.

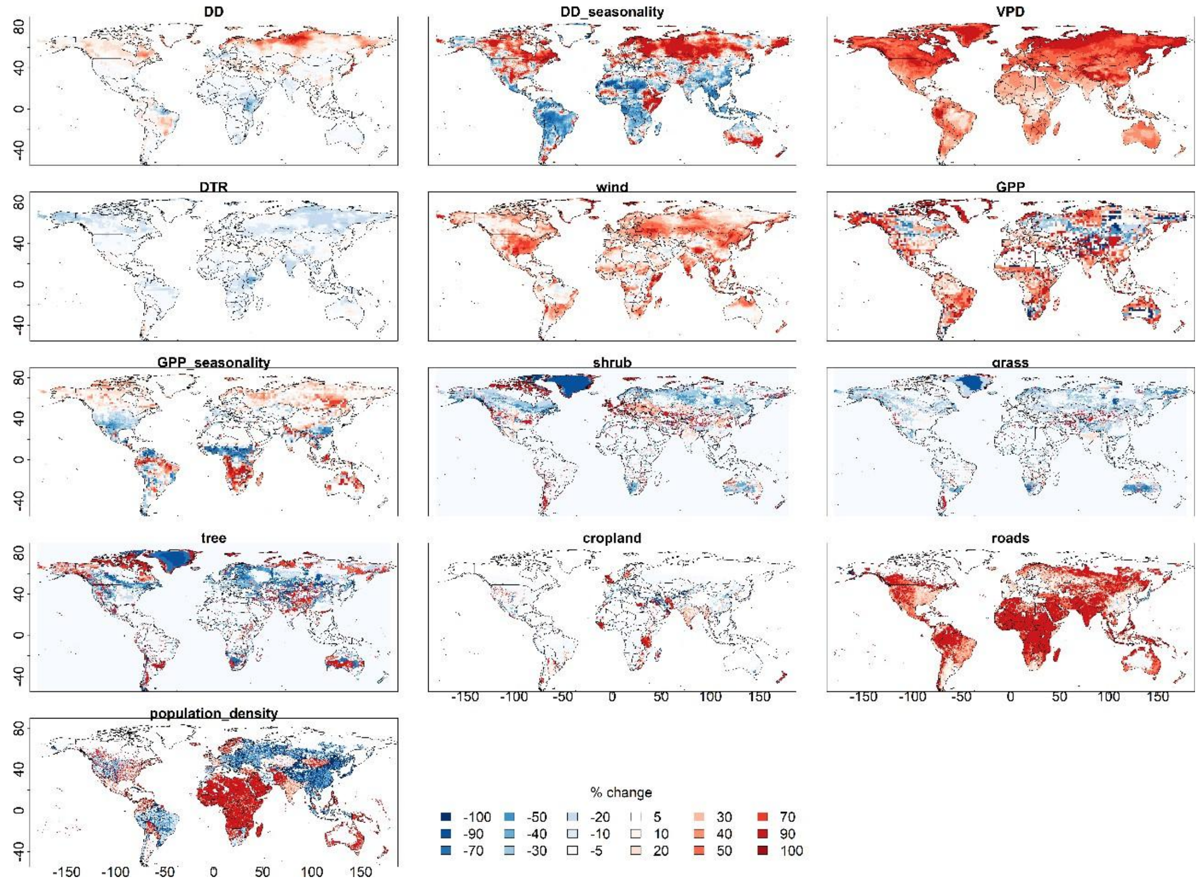

Supplementary Fig S30. **Percentage change in input variables for IPSL-CM5A-LR (low climate change mitigation).** Percentage change in all predictors between the modern-day and the end of century low climate change mitigation scenarios for IPSL-CM5A-LR climate model outputs.

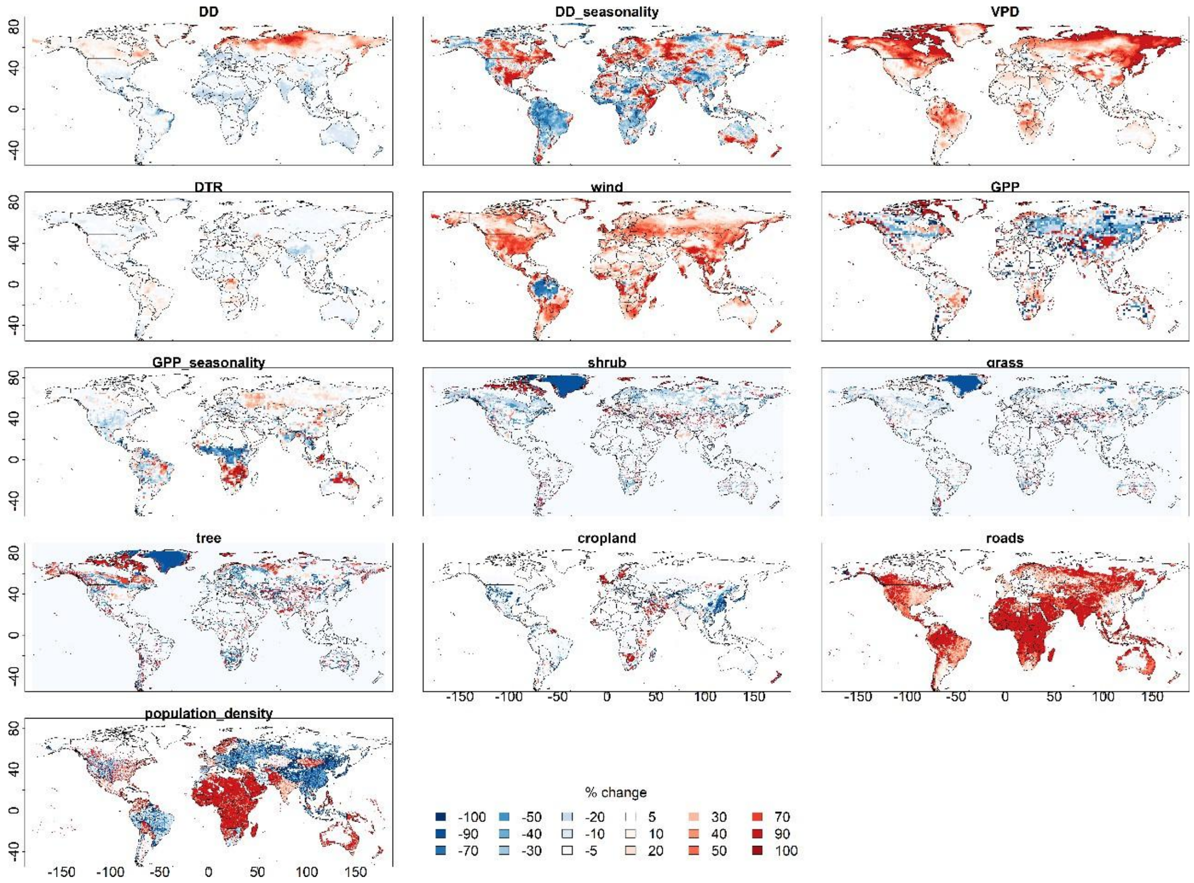

Supplementary Fig S31. Percentage change in input variables for HadGEM2-ES (high climate change mitigation). Percentage change in all predictors between the modern-day and the end of century high climate change mitigation scenarios for HadGEM2-ES climate model outputs.

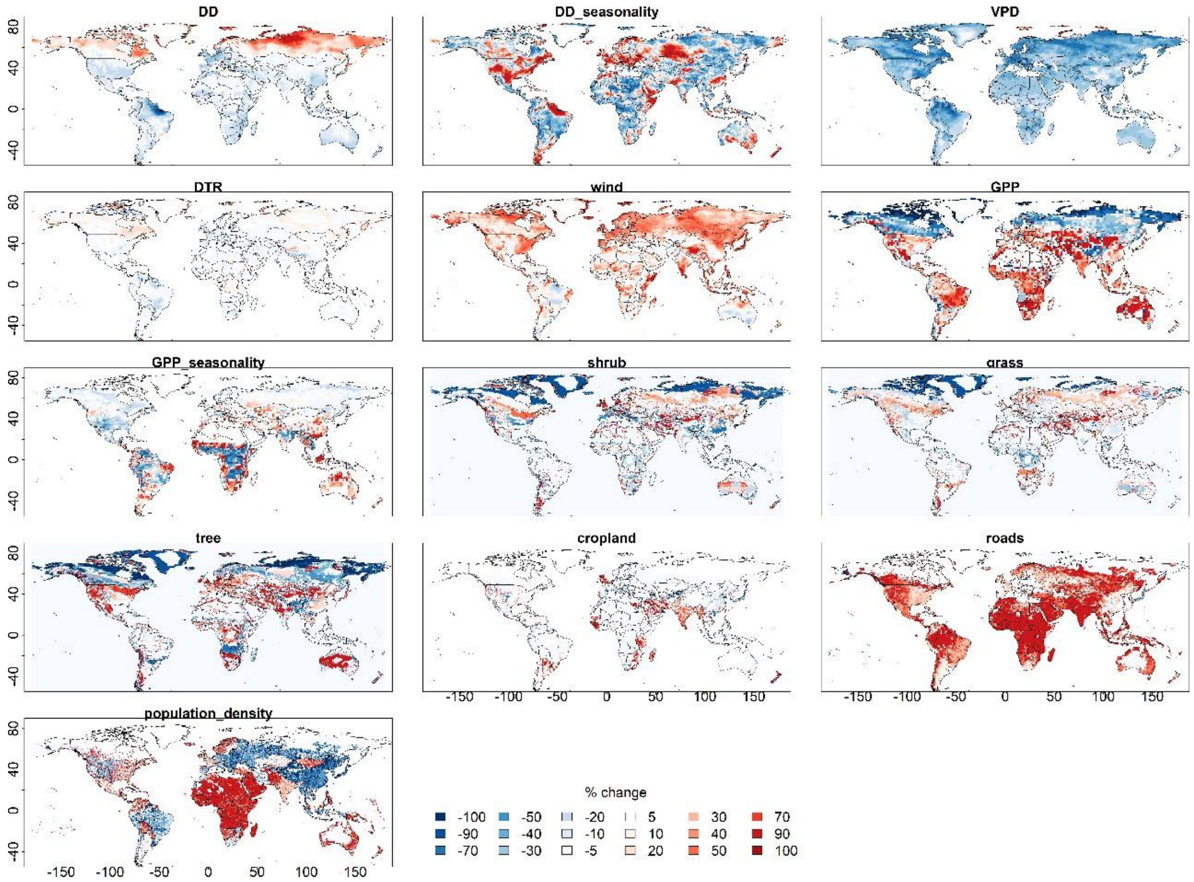

Supplementary Fig S32. **Percentage change in input variables for HadGEM2-ES (low climate change mitigation).** Percentage change in all predictors between the modern-day and the end of century low climate change mitigation scenarios for HadGEM2-ES climate model outputs.

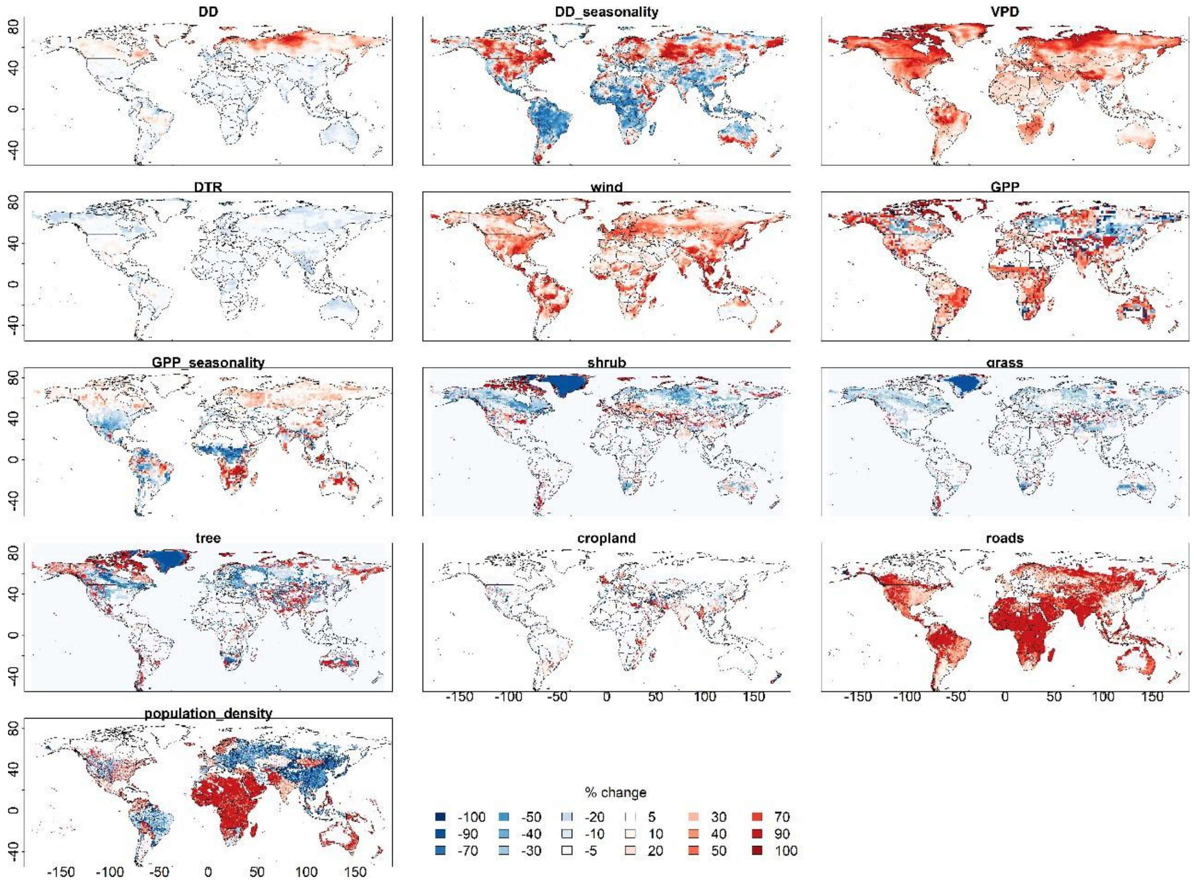

Supplementary Fig S33. Percentage change in input variables for MIROC (high climate change mitigation). Percentage change in all predictors between the modern-day and the end of century high climate change mitigation scenarios for MIROC5 climate model outputs.

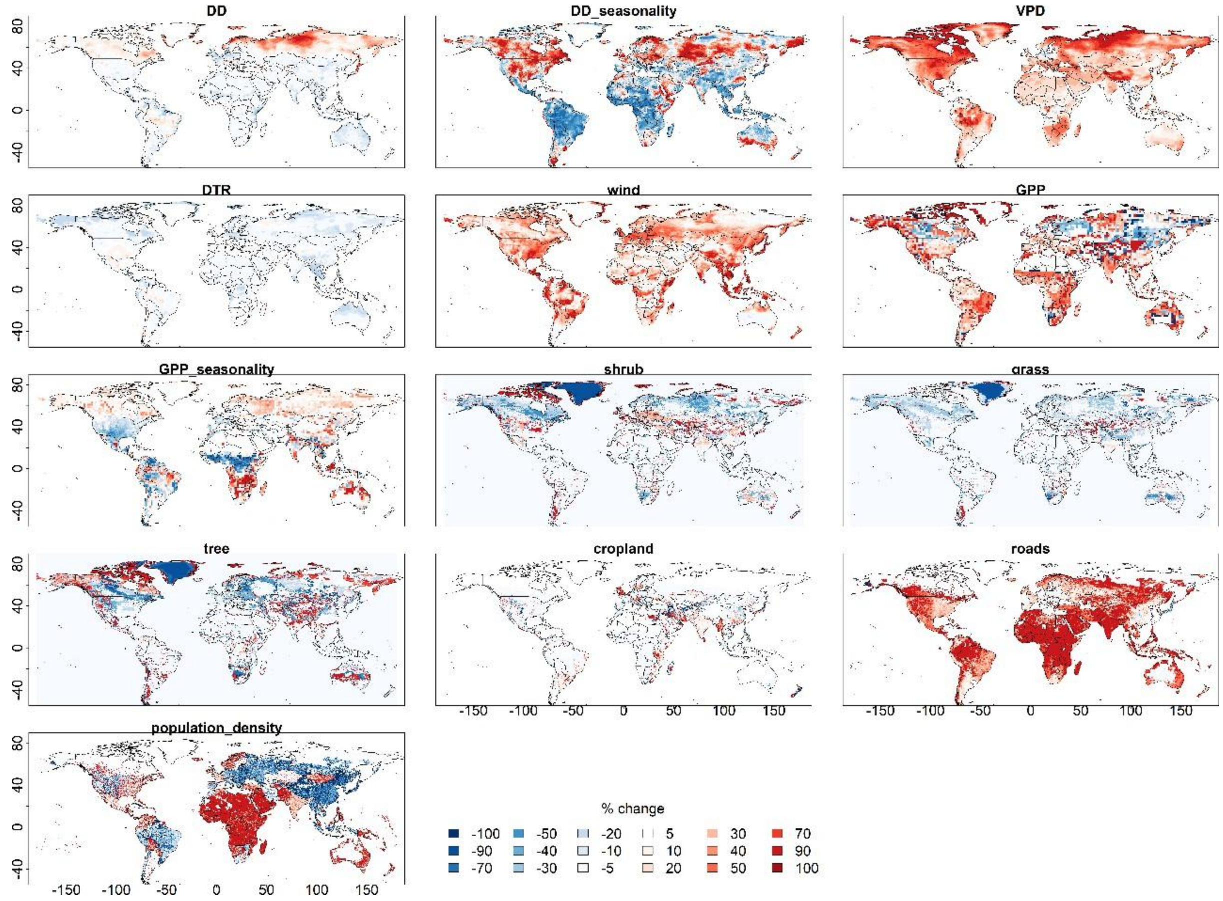

Supplementary Fig S34. **Percentage change in input variables for MIROC5 (low climate change mitigation).** Percentage change in all predictors between the modern-day and the end of century low climate change mitigation scenarios for MIROC5 climate model outputs.
